# Consequences of HIV infection in the bone marrow niche

**DOI:** 10.3389/fimmu.2023.1163012

**Published:** 2023-07-11

**Authors:** Candice Lee Herd, Juanita Mellet, Tsungai Mashingaidze, Chrisna Durandt, Michael Sean Pepper

**Affiliations:** Institute for Cellular and Molecular Medicine, Department of Medical Immunology, South African Medical Research Council (SAMRC) Extramural Unit for Stem Cell Research and Therapy, Faculty of Health Sciences, University of Pretoria, Pretoria, South Africa

**Keywords:** bone marrow niche, HIV, haematopoiesis, haematopoietic stem/progenitor cell, dysregulation

## Abstract

Dysregulation of the bone marrow niche resulting from the direct and indirect effects of HIV infection contributes to haematological abnormalities observed in HIV patients. The bone marrow niche is a complex, multicellular environment which functions primarily in the maintenance of haematopoietic stem/progenitor cells (HSPCs). These adult stem cells are responsible for replacing blood and immune cells over the course of a lifetime. Cells of the bone marrow niche support HSPCs and help to orchestrate the quiescence, self-renewal and differentiation of HSPCs through chemical and molecular signals and cell-cell interactions. This narrative review discusses the HIV-associated dysregulation of the bone marrow niche, as well as the susceptibility of HSPCs to infection by HIV.

## Introduction

The existence of the bone marrow niche was first proposed by Schofield (1) as a specialised microenvironment for the maintenance of haematopoietic stem and progenitor cells (HSPCs), and can be found in the marrow of long bones, vertebrae and iliac crest. The presence of multiple niches within the bone marrow has been proposed due to the presence of distinct subsets of HSPCs in close proximity to non-haematopoietic cell types (2). The bone marrow niche consists of bone matrix and various non-haematopoietic cells, including endothelial cells, stromal cells, neuronal cells and adipocytes (3). Cells of the niche contribute directly to HSPC quiescence, tethering in the bone marrow, homing to niche regions and mobilisation into the circulation, as well as differentiation through intercellular contact and paracrine signalling (3–5).

The bone marrow niche is separated into endosteal (6–8) and perivascular regions (4, 5, 9), each thought to serve a distinct function in the maintenance and mobilisation of HSPCs. Osteolineage cells, perivascular mesenchymal stromal/stem cells (MSCs), CXC chemokine ligand (CXCL)12-abundant reticular (CAR) cells and endothelial cells produce chemoattracting gradients of CXCL12 (also known as stromal-derived factor 1 (SDF-1) and stem cell factor (SCF)) which draw HSPCs to both the endosteal and perivascular regions (4, 5, 7). Non-myelinating Schwann cells and megakaryocytes activate transforming growth factor beta (TGF-β) which has been implicated in maintaining quiescence (5). Megakaryocytes are also thought to contribute to the niche function by releasing CXCL4 and small amounts of thrombopoietin (TPO) which encourage quiescence (4). Coupled with long-range and short-range cytokines regulating haematopoiesis, signalling networks in the bone marrow are extremely complex and have not been fully elucidated. The process of haematopoiesis is well studied and involves many cytokines, chemokines, cell-to-cell interactions and extracellular matrix interactions. However, the *in vivo* functionality, frequency and longevity of HSPCs in humans has not been fully defined.

**Figure 1:**
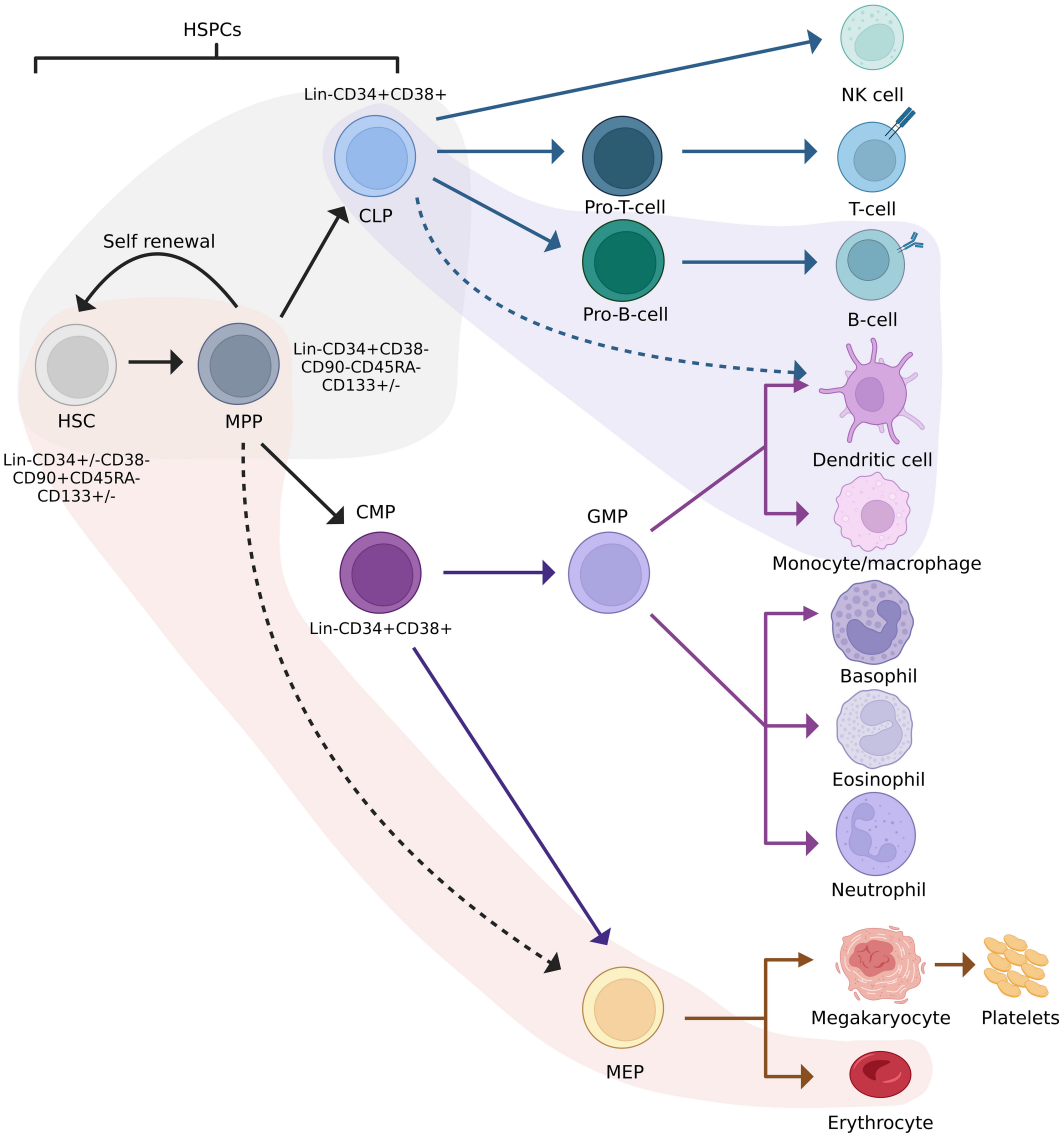
Revised model of haematopoiesis including HSPC phenotypes. Red and purple streams indicate lineage preferences. The grey area indicates cells between which lineage commitment may be reversed. HSC, haematopoietic stem cell; HSPC, haematopoietic stem/progenitor cell; MPP, multipotent progenitor; CLP, common lymphoid progenitor; CMP, common myeloid progenitor; GFM, granulocyte-macrophage progenitor; MEP, megakaryocyte-erythrocyte progenitor. Adapted from Velten et al., 2017, Brown et al., 2018, and Liggett & Sankaran, 2020. Figure created in BioRender.com.

The classical model of haematopoiesis is represented by a hierarchical structure with long-term HSPCs at the apex of the hierarchy (10–12). These cells possess self-renewal capabilities and give rise to short-term HSPCs with limited self-renewal capabilities. Short-term HSPCs differentiate to form multipotent progenitors (MPP), which are precursors of common lymphoid and myeloid progenitors (CLPs/CMPs). MPPs are not able to self-renew but are capable of full lineage differentiation (13). Progeny of CLPs differentiate into lymphoid and natural killer (NK) cells, while progeny of CMPs form granulocyte–macrophage progenitors (GMP) or megakaryocyte–erythrocyte progenitors (MEP). These differentiate into granulocytes and macrophages, and erythrocytes and megakaryocytes, respectively (14). Recent studies suggest that haematopoiesis is more complex than the classical model makes provision for, which includes myeloid-restricted progenitors with long-term repopulating potential (15) and HSPCs expressing platelet-biased genes while having the ability to self-renew (16). These examples represent only a fraction of the data demonstrating the non-classical differentiation potential of HSPCs. Technological advances in the past decade have resulted in a revised model depicted in **Figure 1**.

Since the discovery of the human immunodeficiency virus (HIV) in the early 1980s (17–20), it has spread globally, infecting more than 38 million people worldwide according to the latest available statistics (21). The ability of the virus to evade the immune system (22) and escape antiretroviral therapy (ART) pressure (23) contributes to its persistence *in vivo*. Drug escape and immune evasion are achieved through the action of viral proteins (24–26), immune dysregulation as an indirect consequence of infection (27–30), as well as the high mutation rate of the virus conferred by the low-fidelity HIV reverse transcriptase enzyme (31–33). The majority of HIV infections are caused by HIV-1 group M strains (34), with HIV-1 subtype B (HIV-1B) and C (HIV-1C) claiming 11% and 48% of worldwide infections, respectively (35). Most HIV-1 infections in India, southern Brazil (36) and sub-Saharan Africa are due to HIV-1C (37), while HIV-1B is confined to high income regions such as North America, Europe and Australia (37–40). Despite the prevalence of HIV-1C, HIV-1B dominates the research landscape. Variation between subtypes has been documented for phenotypic properties such as co-receptor tropism (41–45), replication rate and disease progression (45–50), transmission mechanics (51–54), and mutation patterns (55–57). Furthermore, reverse transcription (58) and the emergence of drug resistance (59–62) have been reported to vary between subtypes.

HIV primarily infects cells of the immune system that express cluster of differentiation (CD) 4, C-C-motif chemokine receptor type 5 (CCR5) and C-X-C-motif chemokine receptor type 4 (CXCR4), including CD4+ T-cells and monocyte/macrophages (63); in the case of the former, this results in the depletion of CD4+ cells. The viral reservoir is made up of latently-infected cells which harbour proviral DNA but do not produce viral particles (64). Once established, the reservoir is the most challenging barrier to curing HIV. Latency is complex and regulated at several levels, reviewed elsewhere (64–67). Activation of viral production from latently-infected cells contributes to viral persistence throughout the lifetime of an infected individual. This is evidenced through lineage-tracing which has shown the resurgence of sequences that were dominant during early infection, in the later stages of infection (68–74).

HIV has several concomitant effects once an individual becomes infected. Direct infection of cells with HIV is not the only cause of blood cell depletion, referred to as cytopenia, in HIV patients. Multifactorial, indirect effects associated with HIV infection can also cause cytopenia and other haematological abnormalities. A plethora of cytopenias may present in HIV patients including leukopenia, lymphopenia, anaemia, neutropenia, thrombocytopenia, and pancytopenia (10, 75–81). In a large study conducted in Beijing, neutropenia, thrombocytopenia, and anaemia were partially restored in ART-naïve patients following initiation of treatment (76). However, multiple factors influence restoration of cytopenia following induction of ART (82) including concomitant infections, viral load, tropism and drug resistance, and individual response/adherence to treatment. With the exception of lymphopenia, HIV-associated cytopenias cannot be explained by the lytic cycle of HIV infection. It is unclear whether the haematological abnormalities observed in HIV-infected individuals are due to direct or indirect effects of infection on HSPCs. Various studies suggest these cytopenias may be attributed to disruption of the bone marrow niche housing HSPCs, which maintain the continuous production of blood and immune cells throughout life (83–85). This review will discuss both these possibilities in detail.

## Indirect effects of HIV on HSPCs

**Figure 2:**
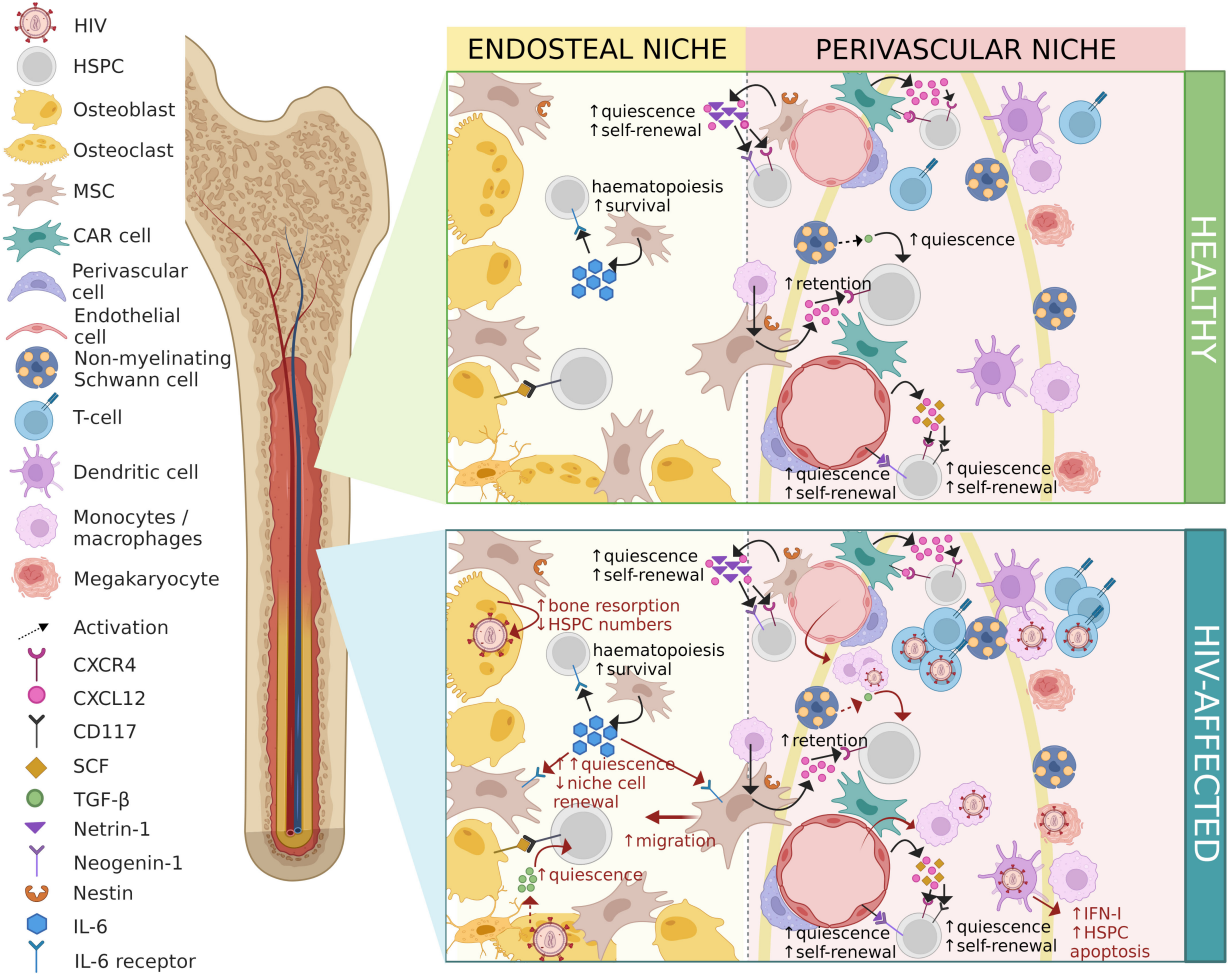
Contrasting healthy and HIV-affected bone marrow niches. HIV-associated changes are shown in red. HIV, human immunodeficiency virus; HSPC, haematopoietic stem/progenitor cell; MSC, mesenchymal stem/stromal cell; CAR cell, CXCL12-abundant reticular cell; CXCR4, C-X-C-motif chemokine receptor type 4; CXCL12, C-X-C-motif chemokine ligand 12; CD117, cluster of differentiation molecule 117; SCF, stem cell factor; TGF-β, transforming growth factor-beta; IL-6, interleukin-6; IFN-I, interferon type-I. Figure created in BioRender.com.

The bone marrow is considered a primary and secondary lymphoid organ allowing for continuous interactions of immune cells (86). The indirect effects of HIV on HSPCs may stem from infection of bone marrow niche cells (87), the effects of HIV proteins on bone marrow cells, or dysregulation of the cytokine milieu which is instrumental in orchestrating dynamic physiological processes including haematopoiesis. The effects of HIV infection on bone marrow niche cells and the consequences for haematopoiesis are described in detail below and are illustrated in **Figure 2**.

### Perivascular niche

The perivascular region around blood vessels that permeate the bone marrow contains perivascular cells, endothelial cells, CAR cells, and nerve fibres including non-myelinated Schwann cells.

Non-myelinating Schwann cells sheath neuronal axons in the perivascular niche of the bone marrow, and participate in niche regulation (88). These cells have been found to activate latent TGF-β released from the bone marrow extracellular matrix (89), and to facilitate circadian regulation of CXCL12 production in Nestin+ MSCs (88). Together, these functions probably account for the maintenance of a quiescent perivascular HSPC pool very closely associated to neuronal axons. Non-myelinating Schwann cell depletion results in reduced HSPC numbers as early as three days post-depletion (89), although the mechanism is unclear. Investigation into HIV-associated neuropathy revealed that the HIV glycoprotein (gp)120 protein stimulated lysosomal exocytosis in Schwann cells (90), releasing axon-exciting adenosine triphosphate (ATP) into the extracellular environment. Exocytosis of lysosomes increased calcium and induced reactive oxygen species (ROS) generation in neighbouring axons, which in turn activates latent TGF-β (91). This could contribute to impaired haematopoiesis by driving HSPCs toward quiescence. Since infection of Schwann cells has only been documented once by electron microscopy (92), HIV-associated neurotoxicity is likely caused by either viral proteins or neurotoxic cytokines released by activated/infected glial cells. The interaction between gp120 and CXCR4 on Schwann cells results in the release of several chemokines, including CC chemokine ligand (CCL)-5 (also known as RANTES) and CXCL1. Release of CCL5 results in the production of TNF-α by dorsal root ganglion neurons and subsequent autocrine neurotoxicity mediated by TNFR1 (93), whereas the release of CXCL1 results in the recruitment of macrophages in mice (94).

Several subtypes of endothelial cells, the cells which line blood vessels, have been identified in the bone marrow, and the distinct functions of each subtype are still being elucidated. Arteriolar, sinusoidal, and endothelial cells expressing endoglin (CD105) are among these. Netrin-1 is expressed by arteriolar endothelial cells and binds to the receptor Neogenin-1 on HSPCs, which is correlated with quiescence and self-renewal of HSPCs *in vivo* (95). Furthermore, arteriolar endothelial cells have been found to produce the majority of endothelial cell-derived SCF, in addition to producing CXCL12. Knock-out of *SCF* in arteriolar endothelial cells results in reduced CD150+CD48-Lin-Sca-1+c-Kit+ primitive HSPCs in mice (96, 97). In bone marrow injury such as irradiation or chemotherapy, an endothelial cell population expressing endoglin (CD105) produces interleukin (IL)-33, which expands umbilical cord blood-derived CD34+ HSPCs *in vitro* and promotes angiogenesis and osteogenesis for bone marrow regeneration (98). Work on the cytokine profiles produced by different endothelial subtypes is lacking. Previous studies in the 1990s showed that endothelial cells produce messenger RNA (mRNA) for cytokines supporting and inhibiting haematopoiesis (99, 100). Cytokines supporting haematopoiesis include granulocyte-macrophage colony-stimulating factor (GM-CSF), IL-1, IL-7, IL-6, TGF-β, IL-8, and IL-11, while thymosin-β4 is a small molecule that inhibits haematopoiesis (100). Endothelial cells also produce mRNA for macrophage inflammatory protein (MIP)-2, platelet-derived growth factor (PDGF), merozoite surface protein (MSP)-1, interferon (IFN)-γ, IL-13 and inhibitin (100). However, the relationship between cytokine mRNA and protein production by endothelial cells for these cytokines is unclear.

In a study comparing bone marrow microvascular endothelial cells from HIV seropositive to those from healthy uninfected donors, these cells have been shown to be permissive to HIV infection *in vivo* (101). HIV-infected microvascular endothelial cells expressing von Willebrand Factor (vWF) were found to produce the HIV protein p24 in long-term culture. Endothelial cells from HIV seropositive donors showed a significant reduction in IL-6 and GM-CSF production in response to IL-1α stimulation compared to uninfected controls (101). In addition to being susceptible to productive HIV infection *in vivo*, HIV proteins contribute to endothelial cell activation, apoptosis, and conversely, stimulate angiogenesis, proliferation, and migration of endothelial cells through various mechanisms (102, 103).

The HIV trans-activator of transcription (Tat) protein induces apoptosis in endothelial cells, but also induces the release of IL-6 and the expression of adhesion markers E-selectin, intracellular adhesion molecule (ICAM)-1, vascular cell adhesion molecule (VCAM)-1, and endothelial leukocyte adhesion molecule (ELAM)-1 which recruit monocytes and increase their migration across the endothelial barrier, increasing monocyte tissue pervasion (104–106). The HIV negative factor (Nef) protein induces apoptosis in endothelial cells by increasing ROS production, causing oxidative stress and cell death (107–111) as well as increasing production of monocyte attractant protein (MCP)-1 (109). The HIV matrix protein p17, similar to Tat, promotes angiogenesis (112) and increases monocyte chemoattractant protein (MCP)-1 production in endothelial cells, as does Nef (113, 114). The implications of these contrasting consequences of HIV infection on endothelial cells *in vivo* remain to be resolved.

CXCL12 is primarily produced by CAR cells (115, 116) and osteoblasts (117) in the bone marrow, and is involved in the homing of cells expressing the cell-surface marker CXCR4 (118, 119), thereby acting as a potent chemoattractant for HSPCs and their progeny in the bone marrow. In addition to chemoattraction, CXCL12-CXCR4 interactions provide physical tethering of CD34+ HSPCs to cell-surface CXCL12 on CAR cells (116, 120). The CXCR4-CXCL12 axis has been found to be critical for the maintenance of the primitive HSPC pool, which is diminished in CXCR4 knock-down mice (116). Neither direct nor indirect effects of HIV infection have been reported for CAR cells to date. There is ongoing debate as to whether a variant in the untranslated region of CXCL12 (designated 3’A) is protective against HIV infection and delays disease progression or whether it is associated with susceptibility to HIV infection and faster progression to AIDS (121).

Dendritic cells and macrophages are both present in the bone marrow stroma in the perivascular niche forming so-called “immune pockets”, where B- and T-lymphocytes are localised. While bone marrow dendritic cells do not play an appreciable role in HSPC maintenance or haematopoiesis, selective ablation of dendritic cells results in increased HSPC mobilisation through an indirect mechanism involving CXCR2 (122). Dendritic cells and macrophages are capable of sustaining HIV replication and could contribute to viral dissemination in the niche (123–125). The perivascular niche is also home to megakaryocytes, which have been found to be susceptible to HIV infection both *in vitro* (126–128) and *in vivo* (128, 129). This relates primarily to viral production and release in the bone marrow and could contribute to thrombocytopenia in HIV patients through the loss of megakaryocytes as a consequence of viral replication.

Circulating monocytes are recruited to tissues and differentiate into tissue-resident macrophages, where they fulfil critical functions in tissue homeostasis. Most of the research on bone marrow macrophages to date has involved murine studies, although whether the findings translate to human bone marrow is not clear. Two populations of bone marrow macrophages have been identified in mice, so-called “osteomacs” present in the endosteal niche in close contact to osteoblasts and Nestin+ MSCs (130), and CD169+ macrophages located in the perivascular niche around Nestin+ MSCs (131). Osteomacs, through cell-to-cell contact, increase Nestin+ MSC production of prostaglandin (PG)-E_2_, which in turn stimulates oncostatin M release by osteomacs, resulting in increased osteoblast mineralisation and differentiation (132). Higher levels of PGE_2_ correlated with increased release of anti-inflammatory IL-10 (133, 134) by macrophages (135). Depletion of CD169+ bone marrow macrophages severely impaired HSPC retention in the niche, and this was associated with a reduction in Nestin+ MSC expression of CXCL12 and SCF mRNA (136, 137). Bone marrow macrophages have been reported to be permissive to HIV infection, although the cytokine profile did not appear to be altered *in vitro* following infection with a number of HIV isolates (138). Whether HIV infection occurs predominantly in monocytes prior to tissue specification into macrophages or in tissue-resident macrophages is difficult to establish. Macrophages may be an important tissue reservoir for HIV capable of sustaining HIV infection *in vivo* (87, 139–142) and their presence would therefore form a large part of the barrier to HIV eradication. Conversely, other researchers have suggested that macrophages play a limited role in HIV replication (143–145). While these cells can be infected by HIV, they may not effectively support viral replication and production. As a result, their contribution to viral spread and long-term persistence in the body may be minimal. Although drawing parallels between murine and human bone marrow is beyond the scope of this review, it is plausible that macrophage depletion resulting from direct HIV infection may result in reduced HSPC retention in the niche and thereby contribute to impaired haematopoiesis in humans.

### Endosteal niche

The endosteal niche is in close proximity to the endosteum of the bone marrow niche which is made up of osteoclasts, osteoblasts, and MSCs. Osteolineage cells (osteoblasts and osteoclasts) were some of the first cells shown to interact with HSPCs and play an important role in HSPC fate. MSCs destined to become osteoblasts occupy the endosteal surface of flat and trabecular bones between the bone and the bone marrow.

Bone marrow MSCs, initially thought to be fibroblasts (146–148), are a heterogenous population of cells forming part of both the perivascular and endosteal stromal cell populations. In addition to replacing osteoblasts and adipocytes as a normal part of cell turnover, bone marrow MSCs play an important role in immunomodulation and HSPC maintenance through cytokine production (149). MSCs constitutively produce IL-6, which is important in haematopoiesis and suppressing the proliferation of MSCs and activated T-cells (149–151). MSCs have also been shown to secrete prostaglandin E_2_ (PGE_2_), which is implicated in the expansion of less primitive HSPCs (152) as well as HSPC recovery and repopulation after chemotherapy (153, 154). In the perivascular niche, periarteriolar MSCs produce Netrin-1 (95) similar to arteriolar endothelial cells, thereby contributing to HSPC quiescence and self-renewal. MSC heterogeneity in the bone marrow is much better described in mice than in humans, and this includes single-cell resolution as has been extensively reviewed elsewhere (155, 156). In murine bone marrow, Lepr+ MSCs enriched for adipocyte and osteoblastic precursors secreting SCF (157) and CXCL12 (116) have been described, which are implicated in HSPC self-renewal (158) and quiescence, respectively, have been described. Although LEPR(hi)CD45(low) BM-MSCs were recently identified in human marrow, the function of these cells remains poorly described (159). Nestin+ MSCs are typically periarteriolar and were found to be clustered around nerve fibres where they produce CXCL12 (156). Characterisation of human bone marrow MSC heterogeneity at the single-cell level remains crucial to elucidating the complex niche dynamics supporting HSPCs, haematopoiesis, as well as immunomodulation in the bone marrow.

While the susceptibility of bone marrow MSCs to HIV infection has not yet been conclusively determined (160), more studies suggest low levels of productive infection - referring to the production of new virus particles (161–163) than studies which demonstrate resistance to infection (138). However, exposure to HIV proteins has varying effects on the differentiation of MSCs. HIV Tat and Nef proteins reduce MSC proliferation and differentiation, and encourage senescence, corresponding to increased oxidative stress and mitochondrial dysfunction (164). Tat increases nuclear factor kappa-light-chain enhancer of activated B cells (NF-κB) activity and inflammatory cytokine secretion, while Nef reduces autophagy, and it was found that the effects of Tat and Nef are cumulative (164). NF-κB expression also drives IL-6 production in MSCs (149), resulting in increased MSC senescence which could explain dysregulation of both bone and fat metabolism in HIV patients. Regulator of expression of virion (Rev) and p55-gag protein expression results in temporal and quantitative changes in key osteo- and adipogenic signals, hampering differentiation (165). Expression of both Rev and p55-gag increase alkaline phosphatase activity and decrease lipid levels, where Rev increases calcium deposition in non-differentiating MSCs. Rev also increases potent peroxisome proliferator-activated receptor gamma (PPAR-γ) expression which drives adipogenic differentiation, and Runt-related transcription factor (RUNX)2 which drives osteogenic differentiation in non-differentiating MSCs (165). In a study assessing the effects of HIV proteins on human MSCs and osteoblast cell lines, HIV proteins p55 and gp120 reduced calcium deposition, alkaline phosphatase activity, and key bone remodelling proteins (166). In contrast, the HIV Rev protein augments MSC osteogenesis (166). HIV gp120 improves adipogenic differentiation, impairs endothelial differentiation, and induces apoptosis of vessel wall-derived MSCs (161). These findings support a role for MSCs in haematopoietic abnormalities resulting from HIV-associated bone marrow niche dysregulation.

Osteoclasts are large, multinucleated monocyte/macrophage-derived cells responsible for resorption of the bone matrix produced by osteoblasts in the continuous, dynamic process of bone remodelling (167, 168). Osteoblasts are smaller osteolineage cells derived from MSCs and produce macrophage colony-stimulating factor (M-CSF) for osteoclastogenesis, osteopontin for continuous formation of bone matrix, as well as a variety of bone marrow niche regulatory cytokines (169). These include IL-6, MIP-1α, SCF, CXCL12, granulocyte colony-stimulating factor (G-CSF), TPO, angiotensin-1, and annexin 2 (169). The bone matrix maintained by osteoclasts and osteoblasts results in a protected environment for long-term HSPCs. Bone remodelling results in the release and activation of TGF-β stored in the bone matrix (168), and a calcium gradient (170), both of which contribute to the quiescence of HSPCs in the endosteal niche. Immature osteoblasts release CXCL12, a potent chemoattractant for cells such as HSPCs which express CXCR4 (169), drawing them towards the endosteum where they bind to SCF on mature osteoblast cell surfaces through the CD117 receptor (169). Angiotensin-1 (171) and TPO (172) produced by osteoblasts also assist in maintaining HSPC quiescence in the endosteal niche.

In HIV infection, bone resorption is increased due to the stimulation of osteoclastogenesis by HIV proteins and direct infection of osteoclasts (173–175). This increased osteoclast activity has been associated with reduced HSPC numbers in the bone marrow (176). In addition to propagating virus through replication, osteoclasts have been implicated in cell-to-cell transmission of HIV-1 between cells of the bone marrow niche (177). Osteoblasts are reportedly not susceptible to HIV infection *in vitro* (178), but respond deleteriously to the presence of HIV proteins. Alkaline phosphatase activity, receptor activator of nuclear factor kappa-B ligand (RANKL) secretion, and calcium (Ca^2+^) deposition by osteoblasts have been reported to be impaired in the presence of HIV p55-gag and gp120 proteins (179). Studies on osteoclasts have produced conflicting findings regarding the induction of apoptosis versus proliferation following exposure to HIV-1 gp120 (180, 181). Degradation of the bone matrix during bone resorption releases and activates an excess of TGF-β in the endosteal niche (182). In addition to stimulating and recruiting MSCs from the perivascular niche to the endosteal niche, high levels of active TGF-β induce quiescence in primitive HSPCs (91), promote proliferation and differentiation of myeloid-primed HSPCs, and hinder lymphoid-primed HSPCs (183). Bone resorption, reduced osteoblast activity in the presence of HIV proteins, and impaired differentiation of MSCs into osteoblasts all contribute to loss of bone density and overall osteopenia observed in HIV patients (184, 185). Consequently, reductions in IL-6, MIP-1α, SCF, CXCL12, G-CSF, and TPO usually produced by osteoblasts are expected in HIV patients. Bone disease in HIV-infected individuals suggests that some of the *in vitro* findings may be transferable (186), although anti-retroviral therapy (ART) has also been implicated (185–187).

Productive infection of bone marrow niche cells would result in the release of HIV proteins which consequently would have an adverse effect on the infected cell and surrounding cells which in turn would negatively affect HSPCs.

### Infiltrating/circulating cells

The bone marrow is highly vascularised, allowing the trafficking of cells and chemical signals to and from the bone marrow through the circulation. In addition to localised effects of HIV on bone marrow cells, altered cytokine profiles produced by trafficked cells affect the niche microenvironment. Monocytes originating from the bone marrow enter the circulation and differentiate into macrophages or dendritic cells in tissues, where they may become infected with HIV (188). Upon infection and pathogen-associated molecular pattern (PAMP)/toll-like receptor (TLR)-initiated migration to secondary lymphoid tissues, dendritic cells are involved in cell-to-cell transfer of HIV to T-cells (189–191). In addition to perpetuating viral transmission, activation of PAMP triggers type I IFN production by dendritic cells (192–195) and the cascade towards chronic immune activation observed in HIV patients (196, 197). Acute type I IFN exposure has been shown to induce proliferation of c-Kit^+^ HSPCs in mice, whereas chronic type I IFN treatment led to HSPC apoptosis due to proapoptotic induction by IFN exposure irrespective of duration (198). The presence of HIV-infected dendritic cells in the bone marrow therefore contributes to haematopoietic dysfunction by directly affecting HSPCs (199).

While monocyte-derived tissue-resident macrophages do not return to the bone marrow, macrophages support viral replication and are important members of the viral reservoir (200) in combination with circulating CD4+ T-cells (201). Circulating CD4+ T-cells are present at higher rates in the bone marrow of HIV positive individuals compared to HIV negative individuals (202), exacerbating the effects of aberrant T-cell cytokine production on the bone marrow. During HIV infection, increased production of IL-4 by T-helper 2 (Th2) CD4+ T-cells was observed (203), which was reported to impair megakaryocyte production in leukaemia (204) and could reasonably be expected to contribute to thrombocytopenia.

It has been suggested that a population of resident memory T-cells in the bone marrow niche may play a role in long-lived immunity against systemic pathogens (205–208), although this is not yet fully understood (209, 210). Resting CD4+ memory and T-cells are well-described as an important latent reservoir for HIV, extensively reviewed elsewhere (206, 211). Bone marrow CD4+ memory T-cells were found to harbour similar levels of virus to circulating CD4+ T-cells in simian immunodeficiency virus (SIV)-infected rhesus macaques (212) and HIV-infected individuals (211). The susceptibility of these cells to HIV infection and their resident status in the bone marrow presents a source of HIV and HIV proteins in the bone marrow outside of circulating infected cells. Activation of this latent reservoir in the bone marrow could result in an increase in HIV load in the bone marrow, which may infect surrounding cells and cause dysregulation of bone marrow niche cells as a consequence of the presence of HIV proteins.

Interactions between cells in the bone marrow are critical for normal haematopoiesis as well as HSPC maintenance and regulation. Bone marrow homeostasis is disrupted during HIV infection as a consequence of direct infection of niche cells and/or the effects of HIV proteins. As a consequence of HIV infection or the effects of HIV proteins on perivascular niche cells, perivascular niche HSPCs are driven towards quiescence and mobilisation, thereby impairing haematopoiesis. Endosteal niche HSPCs are driven to mobilisation due to the breakdown of normal bone remodelling and MSC senescence resulting from HIV infection. The consequences of the indirect effects of HIV infection on HSPCs are therefore deleterious, ultimately reducing the number of HSPCs in the bone marrow and the creation of a quiescence-supporting environment for the remaining HSPCs.

## Direct effects of HIV on HSPCs

The indirect effects of HIV infection on HSPCs are complex and cumulative, contributing to the impairment of HSPC function. The direct effects of HIV on HSPCs encompass both direct infection and the effect of HIV proteins on HSPC function. However, the literature presents opposing conclusions regarding the susceptibility of HSPCs to HIV infection.

HSPCs in the bone marrow are directed to remain quiescent or divide and differentiate, forming blood and immune cells in response to the cytokine milieu. At key points, differentiating HSPCs will become lineage restricted and only form the cell types dictated by the cytokine milieu. This means that one infected HSPC would produce a limited number of haematopoietic cell types harbouring HIV. Nixon et al., 2013 demonstrated that HSPC progeny generated through colony-forming assays of CD34+ cells from HIV-infected humanised mice, harboured HIV. Clonally infected cells resulting from HSPC division and differentiation would therefore be restricted to a single or limited number of haematopoietic cell types, depending on the differentiation potential of the infected HSPC and on external stimulus directing haematopoiesis. Transcriptional activation during differentiation could activate HIV replication from integrated or episomal provirus, possibly resulting in cell death due to the lytic nature of HIV replication. Carter et al., 2010 (213) showed that CD34+ cells expressing HIV gene products were markedly depleted in culture compared to a transduced control, which could indicate some other mechanism of cell death in infected HSPCs. *In vivo*, host cell lysis or death might contribute to the absence of terminally differentiated HIV-infected cells of all haematopoietic lineages harbouring clonal virus initially of HSPC-origin. HIV infection may skew haematopoiesis towards or away from certain lineages (214, 215), which may contribute to cytopenia and the lack of clonal infection of certain haematopoietic cell types. This is not well described in literature as bone marrow research is limited to static snapshots of a highly dynamic environment.

The controversy in literature dates back to the early 1990s with Stanley and colleagues detecting HIV in CD34+ HSPCs from seropositive patients (216) and Neal and colleagues presenting alternate data showing that CD34+ HSPCs were rarely infected with HIV in asymptomatic patients (217). This was followed by a number of studies with different conclusions and one paper suggesting that HIV-1 subtypes may differ in their ability to infect HSPCs (218). Several studies found HSPC subsets to be resistant to HIV infection (217–230), the suggested mechanism being through a p21-mediated pre-integration block (231). Given the inducible expression of HIV proteins in the presence of a pre-integration block, the findings may suggest transient transcription from episomal proviral DNA in HSPCs (213). In contrast, a number of studies have detected HIV in HSPCs (213, 214, 216, 218, 222, 226, 229, 230, 232–236). Carter and colleagues showed latent infection of Lin-CD34+CD133+CD38- primitive HSPC subsets *in vitro* and corroborated these findings with bone marrow CD34+ HSPCs from HIV infected individuals with high viral load (213). However, their findings also suggest that HIV-infected cells actively expressing HIV proteins were short-lived compared to their latently-infected counterparts (213). Several follow-up studies reported similar findings (214, 230, 235, 236), which are presented in **Table 1**. A recent study reported that a small subset of the heterogenous CD34+ HSPC population expresses low levels of CD4, and that this subset was found to harbour HIV genomes *in vivo* (237). While detection of HIV in this subset is not necessarily surprising, the fact that both R5- and X4-tropic HIV genomes were detected was notable as CXCR4 is usually expressed in a greater fraction of CD34+ HSPCs than is CCR5 (213). While HSPC susceptibility to HIV infection hinges largely on the expression of CD4, CXCR4, and CCR5, CD4-independent infection mechanisms have been described (238–241) and should not be discounted for infection of HSPCs. Most recently, Renelt and colleagues made a strong argument for HIV infection of CD133+ and CD34+CD133- HSPC subpopulations in some donors, and their contribution to viremia using proviral sequence tracing (222).

**Table 1:** Comparison of literature on the susceptibility of HSPCs to HIV infection.

HIV DETECTED							
Cells			Infection		HIV detection		Reference
Source	Phenotype	Activation method	HIV	*In vitro/vivo*	Method	Target	
BM	CD34+	NA	Uncharacterised HIV+ donors (Zaire and North America)	*In vivo*	PCR	*env*, *gag*	Stanley et al., 1992 (216)
PB	CD34+/- (BFU-E and CFU-GM colonies)	Overnight pre-stimulation in SCF, IL-3, GM-CSF, Epo	X4-tropic HIV-1B molecular clone	*In vitro*	RT-PCR, ELISA	*tat*, *gag* p24	† Chelucci et al., 1995 (226)
BM	CD34+, CD34+CD38+, CD34+CD38-,	NA	R5- and X4-tropic HIV-1 molecular clones. Uncharacterised patient virus (USA)	*In vitro; in vivo*	PCR, ELISA	*gag, LTR* p24	Shen et al., 1999 (229)
PB, UCB	CD34+, MNCs	Pre-cultured with SCF, GM-CSF, IL-3, Epo	R5-tropic HIV-1C molecular clones and primary HIV+ patients (Botswana)	*In vitro, in vivo*	RT-PCR, ELISA	*gag* p24	† Redd et al., 2007 (218)
BM, UCB	CD34+, CD133+	NA	Uncharacterised patient virus; R5X4-tropic HIV-1B molecular clones and pseudovirus	*In vitro, in vivo*	Flow cytometry, qPCR	Gag (KC57 and anti-p24 mAB), *LTR*	‡ † Carter et al., 2010 (213)
BM, UCB	CD34+, CD133+	Pre-stimulation in SCF, TPO, FLT3-L, IGFBP-2	X4- and R5X4-tropic HIV-1B molecular clones and pseudotyped viruses	*In vitro, in vivo*	Flow cytometry	GFP, IC Gag	‡ Carter et al., 2011 (230)
BM	CD133+, CD34+CD45RA-CD38-	Pre-stimulation in SCF, TPO, FLT3-L, IGFBP-2	Pseudotyped virus	*In vitro*	Flow cytometry	GFP, PLAP, p24	‡ McNamara et al., 2012 (235)
BM	CD133+	NA	Uncharacterised HIV+ donors on ART with plasma viral loads of <48 copies/mL.	*In vivo*	qPCR	*Gag, LTR*	* McNamara et al., 2013 (236)
UCB, fetal liver	CD34+CD38+CD123+ (CMP), CD34+CD38+CD45RA+ (GMP) and CD34+CD38+CD110+ (MEP)	NA	Wild-type viruses were created from proviral plasmids p89.6, pYJRCSF, and pNL4-3.	*In vitro*	qRT-PCR	*Gag, LTR*	‡ Nixon et al., 2013 (214)
PB, BM	Lin-CD34+	NA	Uncharacterised HIV+ donors (naïve and on ART)	*In vivo*	qPCR	*LTR*	* Bordoni et al., 2015 (232)
BM	Lin-CD34+	NA	HIV-infected humanized mice (5 – 14 weeks post-infection)	*In vivo*	qPCR, Immunofluorescence	*Gag*	Araínga et al., 2016 (233)
BM	CD34+, CD133+	UCB-derived cells pre-cultured for 4 days	X4- and R5-tropic HIV-1B molecular clones. Uncharacterised HIV+ donors	*In vitro, in vivo*	Flow cytometry, PCR	*Gag, env*	* Sebastian et al., 2017 (237)
BM	CD133+, CD34+CD133-	NA	Uncharacterised HIV+ donors	*In vivo*	PCR	*Gag*, *env*	* Zaikos et al., 2018 (234)
BM, CB	Lin-, CD34+CD38-CD45RA-Lin-, Lin-CD34+CD38-CD45RA-CD90-, CD34+CD38-CD45RA-CD90+, CD34+CD38+	NA	X4-tropic pseudotyped GFP reporter viruses, X4- and R5-tropic HIV-1B molecular clones. Uncharacterised HIV+ donors, one donor with confirmed HIV-1B infection	*In vitro; in vivo*	Flow cytometry, qPCR	GFP, p24, *HIV-1 R-U5/gag*	* Renelt et al., 2022 (222)
HIV NOT DETECTED							
Cells			Infection		HIV detection		Reference
Source	Phenotype	Activation method	Virus	*In vitro/vivo*	Method	Target	
BM	Colony-forming cells from T-cell and adherent BM cell-depleted BM fractions	NA	Uncharacterised HIV+ donors (North America); HIV-1B and HIV-2A (isolate ROD)	*In vivo; in vitro*	PCR	*Gag*	Molina et al., 1990 (223)
BM	CD34+	NA	Uncharacterised HIV+ donors (North America)	*In vivo*	PCR	*Env*, *gag*	Davis et al., 1991 (224)
BM	CD34+	NA	Uncharacterised HIV+ donors (France)	*In vivo*	PCR, Flow cytometry	*gag* p24, gp120	Louache et al., 1992 (225)
PB	CD34+/- (CFU-GEMM)	Overnight pre-stimulation in SCF, IL-3, GM-CSF, Epo	X4-tropic HIV-1B molecular clone	*In vitro*	RT-PCR, ELISA	*Tat*, *gag* p24	Chelucci et al., 1995 (226)
BM	CD34+, CD34-, MNCs	NA	Uncharacterised HIV-1+ donors (USA) on ART with no AIDS-defining illness	*In vivo*	PCR	*Gag, pol*	Neal et al., 1995 (217)
BM	CD34+CD38-; CD34+CD4+	NA	Uncharacterised HIV+ donors (France)	*In vivo*	PCR	*gag*	Marandin et al., 1996 (227)
BM	CD34+CD38-; CD34+CD38+	NA	R5 and R5X4-tropic HIV-1 and R5X4 HIV-2 molecular clones	*In vitro*	PCR, ELISA	*gag* p24	Weichold et al., 1998 (228)
BM	G0 CD34+	7-day pre-culture	R5- and X4-tropic HIV-1 molecular clones. Uncharacterised patient virus (USA)	*In vitro; in vivo*	PCR, ELISA	*gag, LTR* p24	Shen et al., 1999 (229)
PB, UCB	CD34+, MNCs	Pre-cultured with SCF, GM-CSF, IL-3, Epo	R5-tropic HIV- 1B molecular clones and primary HIV (USA)	*In vitro; in vivo*	RT-PCR, p24 ELISA	*gag*	Redd et al., 2007 (218)
BM, UCB	CD34+, CD133+	Pre-stimulation in SCF, TPO, FLT3-L, IGFBP-2	R5- tropic HIV-1B molecular clones; pseudotyped viruses; HIV+ donors	*In vitro, in vivo*	Flow cytometry	GFP, IC *Gag*	Carter et al., 2011 (230)
BM	CD34+	Pre-cultured with SCF, TPO, FLT3-L and GM-CSF and TNF-α, or PMA	HIV+ donors (Patients on ART and VL <50 copies/mL)	*In vivo*	PCR	*gag*	Durand et al., 2012 (219)
BM	Lin-CD34+, Lin-CD34-	NA	HIV-1B+ donors (Patients on ART and VL <45-70 copies/mL)	*In vivo*	PCR	Target not specified	Josefsson et al., 2012 (220)
UCB	CD34+	24 hr pre-stimulation with TPO, SCF, and FLT3-L	VSV-G-pseudotyped virus with a modified pNL4.3 HIV-1-based core including an mCherry ORF	*In vitro*	Flow cytometry, qPCR	mCherry	Griffin & Goff, 2015 (221)
BM	Lin-CD34+CD38-CD45RA-CD90-, CD34+CD38-CD45RA-CD90+	NA	R5-tropic pseudotyped GFP reporter viruses	*In vitro*	Flow cytometry	GFP	Renelt et al., 2022 (222)

BM, bone marrow; PB, peripheral blood; UCB, umbilical cord blood; BFU-E, burst-forming unit erythroid; CFU-GM, colony-forming unit granulocyte-macrophage; CMP, common myeloid progenitor; GMP, granulocyte-macrophage progenitor; MEP, megakaryocyte-erythroid progenitor; CFU-GEMM, colony-forming unit granulocyte-erythroid-macrophage-megakaryocyte; SCF, stem cell factor; IL-3, interleukin-3; GM-CSF, granulocyte-macrophage colony-stimulating factor; Epo, erythropoietin; IGFBP-2, insulin-like growth factor binding protein-2; TNF-α, tumour necrosis factor alpha; PMA, phorbol myristate acetate; FLT3-L, fms-like tyrosine kinase receptor 3 ligand; TPO, thrombopoietin; R5, C-C-motif chemokine receptor type 5; X4, C-X-C,motif chemokine receptor type 4; PCR, polymerase chain reaction; qPCR, quantitative PCR; RT-qPCR, real-time qPCR; ELISA, enzyme-linked immunosorbent assay; IC, intracellular; GFP, green fluorescent protein.

*T-cell contamination was robustly excluded from analysis (<1% T-cells).

‡T-cell contamination unlikely due to single-cell HIV-detection by flow cytometry.

†T-cell contamination unlikely due to culture conditions (CFU assays).

A closer inspection of studies exploring the susceptibility of HSPCs to HIV infection outlined in **Table 1** reveals that different experimental approaches may in part explain the lack of consensus between studies to some degree. Variations in culturing, the use of growth factors, HIV moieties, infection strategy, and HIV detection method could contribute to variation between results. These factors and how each could affect the outcome of the study are discussed in more detail below.

HSPCs are rare cells that often require *in vitro* expansion so that enough cells are obtained to optimally perform experiments. Numerous studies have cultured or expanded HSPCs for several days before *in vitro* infection with HIV. Expression of CXCR4 is upregulated on murine HSPCs after overnight incubation (242), which may artefactually increase susceptibility to CXCR4-tropic HIV. Similarly, HIV integration and replication is dependent on the activation state of target cells (243) with dividing cells being more susceptible to productive HIV infection. The majority of HSPCs (>90%) are in a quiescent state *in vivo* (244) in the bone marrow niche; expansion prior to infection would therefore create an *ex vivo* artefactual state. Expansion and culturing of HSPCs *in vitro* is often performed in the presence of haematopoietic cytokines which promote expansion and HSPC survival in culture. However, there is not currently a standardised cytokine cocktail for HSPC expansion and maintenance. Studies investigating the susceptibility of HSPCs to HIV infection have been performed with (218, 226, 230, 235) and without (229, 231, 245) cytokines. The duration of culture and the supplementation of medium with cytokines could therefore result in increased susceptibility of HSPCs to HIV infection that is not inherent but rather an artefact of culturing.

The use of HIV propagated *in vitro* in the form of laboratory-generated HIV molecular clones, pseudo- or pseudo-typed virus, or cultured primary virus has several aspects where outcome-critical variation between studies could occur. The most glaring differences between studies are (i) multiplicity of infection (MOI), (ii) infection strategy, and (iii) nature of virus used. Unrealistic bombardment of target cells with extremely high MOIs, referring to the number of infectious units per target cell, could result in artefactual infection *in vitro*, which is unlikely *in vivo*. The infection strategy is similarly crucial to a translatable experimental outcome. Infection in small volumes or using centrifugal force (termed “spinoculation”) to create close contact between cells are two methods commonly used to increase the potential for infection. Spinoculation is not a physiological condition and could therefore also result in artefactual infection. The nature of the virus used in experiments is constrained by several factors including but not limited to biosafety, availability of comparable research tools, and the effect of HIV proteins on target cells which can affect results. As previously mentioned, HIV-1B (being the most-studied subtype) epitomises what is known about HIV infection. However, distinct characteristics including coreceptor usage during early and late infection (246) and reduced cytopathic effects (58) have been documented for HIV-1C, which could affect research outcomes in terms of latency and host cell susceptibility. Variation from HIV-1B has been reported for non-B subtypes in several aspects related to viral fitness and disease progression (45–48, 50, 51, 55, 58, 59, 61, 218, 247–250) which are outside the scope of this review but are important when comparing research findings.

The method used to detect HIV is another critical factor to be considered when comparing studies. In addition to the increased sensitivity that comes with improvements in detection technologies over time, studies have varied with the technology used to detect the presence of HIV in target cells. Proviral DNA, viral transcripts, or viral proteins can be targeted, and each detection method comes with a limit of detection and considerations for use. This is particularly important where, as with HSPCs, it is reasonable to expect low to very low proportions of infected cells. This is illustrated in a study by Izopet et al. (251) who were able to detect four proviral genomes per million cells and found that the frequency of infection of highly susceptible CD4+ T-cells *in vivo* can be lower than 1%, as reported in other studies (252–254). The sensitivity of the HIV detection method is often not reported, and the improvements of technologies over time are difficult to categorise, but these are equally important to consider when comparing older and more recent studies.

Disruption of HSPC function can also be caused by the presence of HIV proteins. The HIV receptor protein gp120 has been shown to impair the clonogenic potential of HSPCs and induce apoptosis through Fas-dependent endogenous TGF-β upregulation (255). Suppression of HSPC colony formation is caused by HIV-1 p24 (256). Exposure to HIV Tat protein stimulates TGF-β production in macrophages resulting in myelosuppression *in vitro* (255), and viral protein R (Vpr) has been shown to induce phagocytosis of bone marrow cells by mononuclear phagocytes (257). Blocking TGF-β in purified CD34+ HSPCs exposed to HIV reportedly improved growth and survival (255), and this is supported by the simultaneous downregulation of a proliferation-inducing ligand (APRIL) with TGF-β upregulation induced by exposure to gp120 (258). Furthermore, Nef has been shown to act as a PPARγ agonist with deleterious effects on early haematopoiesis in macaques (259). Cumulatively, the effects of HIV proteins in the bone marrow are deleterious to HSPC and niche cell function and survival, and ultimately contribute to haematological abnormalities present in HIV patients independently of direct HSPC infection.

The conditions under which HSPCs may become susceptible to HIV infection in the bone marrow are not clear based on current information from the literature; what is clear however is that HIV proteins have a direct suppressive effect on HSPC function.

## Concluding remarks

Haematopoietic dysfunction in HIV patients is well-documented and results from the combined direct and indirect effects of HIV on HSPCs. The bone marrow niche is a uniquely complex environment which is yet to be fully understood. Healthy human bone marrow is therefore not completely represented in literature, which makes it difficult to fully model the marrow under conditions of HIV infection. The limited understanding of *in vivo* susceptibility of bone marrow cells to HIV and the fact that bone marrow cell types have largely been studied *in vitro* or in animal models contribute to the paucity of literature on the HIV-infected marrow. Moreover, the evolution of HIV detection methods over time, and the understanding that detection of HIV proteins or partial DNA does not necessarily indicate productive infection, compound this challenge. Although it is undeniable that HIV affects haematopoiesis, the susceptibility of HSPCs to HIV has long been debated. Studies investigating HIV infection in HSPCs differ critically in methodology and HSPC subpopulations used. This review has aimed to highlight what is currently known about the consequences of HIV infection on the bone marrow niche, and to summarise the studies to date which have attempted to determine the susceptibility of HSPCs to HIV infection. This is particularly relevant to the fields of stem cell transplantation and HIV pathogenesis, and potentially to the treatment of HIV-associated haematological malignancies.

## Author contributions

CH and JM wrote, compiled, and edited the manuscript. CH made the figures. TM contributed to the manuscript. MP and CD were involved in manuscript conception. MP edited the manuscript. All authors contributed to the article and approved the submitted version.

## References

[1] Schofield R. The relationship between the spleen colony-forming cell and the haemopoietic stem cell. Blood Cells (1978) 4(1–2):7–25. 747780

[2] Beerman I Luis TC Singbrant S Lo Celso C Méndez-Ferrer S. The evolving view of the hematopoietic stem cell niche. Exp Hematol (2017) 50:22–6. doi: 10.1016/j.exphem.2017.01.008 PMC5466495 28189651

[3] Szade K Gulati GS Chan CKF Kao KS Miyanishi M Marjon KD. Where hematopoietic stem cells live: the bone marrow niche. Antioxid Redox Signal (2018) 29(2):191–204. doi: 10.1089/ars.2017.7419 29113449 PMC6016729

[4] Asada N Takeishi S Frenette PS. Complexity of bone marrow hematopoietic stem cell niche. Int J Hematol (2017) 106(1):45–54. doi: 10.1007/s12185-017-2262-9 28534115 PMC8808638

[5] Morrison SJ Scadden DT. The bone marrow niche for haematopoietic stem cells. Nature. (2014) 505(7483):327–34. doi: 10.1038/nature12984 PMC4514480 24429631

[6] Cordeiro-Spinetti E Taichman RS Balduino A. The bone marrow endosteal niche: how far from the surface? J Cell Biochem (2015) 116(1):6–11. doi: 10.1002/jcb.24952 25164953 PMC4229422

[7] Lévesque JP Helwani FM Winkler IG. The endosteal osteoblastic niche and its role in hematopoietic stem cell homing and mobilization. Leukemia. (2010) 24(12):1979–92. doi: 10.1038/leu.2010.214 20861913

[8] Tamma R Ribatti D. Bone niches, hematopoietic stem cells, and vessel formation. Int J Mol Sci (2017) 18(1):151. doi: 10.3390/ijms18010151 28098778 PMC5297784

[9] Yu VWC Scadden DT. Chapter two - hematopoietic stem cell and its Bone marrow niche. In: Bresnick EH, editor. Hematopoiesis. Academic Press (2016). p. 21–44. doi: 10.1016/bs.ctdb.2016.01.009 PMC6854531 27137653

[10] Durandt C Potgieter JC Mellet J Herd C Khoosal R Nel JG. HIV And haematopoiesis. S Afr Med J (2019) 109(8):40–5. doi: 10.7196/SAMJ.2019.v109i8b.13829 31662148

[11] Liggett LA Sankaran VG. Unraveling hematopoiesis through the lens of genomics. Cell. (2020) 182(6):1384–400. doi: 10.1016/j.cell.2020.08.030 PMC7508400 32946781

[12] Brown G Tsapogas P Ceredig R. The changing face of hematopoiesis: a spectrum of options is available to stem cells. Immunol Cell Biol (2018) 96(9):898–911. doi: 10.1111/imcb.12055 29637611

[13] Velten L Haas SF Raffel S Blaszkiewicz S Islam S Hennig BP. Human haematopoietic stem cell lineage commitment is a continuous process. Nat Cell Biol (2017) 19(4):271–81. doi: 10.1038/ncb3493 PMC5496982 28319093

[14] Kondo M. Lymphoid and myeloid lineage commitment in multipotent hematopoietic progenitors. Immunol Rev (2010) 238(1):37–46. doi: 10.1111/j.1600-065X.2010.00963.x 20969583 PMC2975965

[15] Yamamoto R Morita Y Ooehara J Hamanaka S Onodera M Rudolph KL. Clonal analysis unveils self-renewing lineage-restricted progenitors generated directly from hematopoietic stem cells. Cell. (2013) 154(5):1112–26. doi: 10.1016/j.cell.2013.08.007 23993099

[16] Sanjuan-Pla A Macaulay IC Jensen CT Woll PS Luis TC Mead A. Platelet-biased stem cells reside at the apex of the haematopoietic stem-cell hierarchy. Nature. (2013) 502(7470):232–6. doi: 10.1038/nature12495 23934107

[17] Montagnier L Chermann JC Barre-Sinoussi F Klatzmann D Wain-Hobson S Alizon M. Lymphadenopathy associated virus and its etiological role in AIDS. Princess Takamatsu Symp (1984) 15:319–31. 6100650

[18] Barré-Sinoussi F Chermann JC Rey F Nugeyre MT Chamaret S Gruest J. Isolation of a T-lymphotropic retrovirus from a patient at risk for acquired immune deficiency syndrome (AIDS). Sci (80-). (1983) 220(4599):868–71. doi: 10.1126/science.6189183 6189183

[19] Durack DT. Opportunistic infections and kaposi’s sarcoma in homosexual men. N Engl J Med (1981) 305(24):1465–7. doi: 10.1056/NEJM198112103052408 6272112

[20] Masur H Michelis MA Greene JB Onorato I Vande Stouwe RA Holzman RS. An outbreak of community-acquired pneumocystis carinii pneumonia: initial manifestation of cellular immune dysfunction. N Engl J Med (1981) 305(24):1431–8. doi: 10.1056/NEJM198112103052402 6975437

[21] UNAIDS. Global HIV & AIDS statistics [[/amp]]mdash; fact sheet. In: Global HIV & AIDS statistics [[/amp]]mdash; fact sheet (2022). Available at: https://www.unaids.org/en/resources/fact-sheet.

[22] Guha D Ayyavoo V. Innate immune evasion strategies by human immunodeficiency virus type 1. Isrn Aids. (2013) 2013:1–10. doi: 10.1155/2013/954806 PMC3767209 24052891

[23] Günthard HF Calvez V Paredes R Pillay D Shafer RW Wensing AM. Human immunodeficiency virus drug resistance: 2018 recommendations of the international antiviral society-USA panel. Clin Infect Dis (2019) 68(2):177–87. doi: 10.1093/cid/ciy463 PMC6321850 30052811

[24] Frankel AD Young JAT. HIV-1: fifteen proteins and an RNA. Annu Rev Biochem (1998) 67:1–25. doi: 10.1146/annurev.biochem.67.1.1 9759480

[25] Rose KM Marin M Kozak SL Kabat D. The viral infectivity factor (Vif) of HIV-1 unveiled. Trends Mol Med (2004) 10(6):291–7. doi: 10.1016/j.molmed.2004.04.008 15177194

[26] Pereira EA daSilva LLP. HIV-1 nef: taking control of protein trafficking. Traffic. (2016) 17(9):976–96. doi: 10.1111/tra.12412 27161574

[27] Boasso A Shearer GM Chougnet C. Immune dysregulation in human immunodeficiency virus infection: know it, fix it, prevent it? J Intern Med (2009) 265(1):78–96. doi: 10.1111/j.1365-2796.2008.02043.x 19093962 PMC2903738

[28] Vishnu P Aboulafia DM. Haematological manifestations of human immune deficiency virus infection. Br J Haematol (2015) 171(5):695–709. doi: 10.1111/bjh.13783 26452169

[29] Kirchhoff F Silvestri G. Is nef the elusive cause of HIV-associated hematopoietic dysfunction? J Clin Invest (2008) 118(5):1622–5. doi: 10.1172/JCI35487 PMC2323195 18431512

[30] Lederman MM Funderburg NT Sekaly RP Klatt NR Hunt PW. Residual immune dysregulation syndrome in treated HIV infection. Adv Immunol (2013) 119:51–83. doi: 10.1016/B978-0-12-407707-2.00002-3 23886064 PMC4126613

[31] Ji J Loeb LA. Fidelity of HIV-1 reverse transcriptase copying a hypervariable region of the HIV-1 env gene. Virology. (1994) 199(2):323–30. doi: 10.1006/viro.1994.1130 7510083

[32] Cuevas JM Geller R Garijo R López-Aldeguer J Sanjuán R. Extremely high mutation rate of HIV-1 *In vivo* . PloS Biol (2015) 13(9):e1002251. doi: 10.1371/journal.pbio.1002251 26375597 PMC4574155

[33] Roberts JD Bebenek K Kunkel TA. The accuracy of reverse transcriptase from HIV-1. Sci (80-). (1988) 242(4882):1171–3. doi: 10.1126/science.2460925 2460925

[34] Castro-Nallar E Pérez-Losada M Burton GF Crandall KA. The evolution of HIV: inferences using phylogenetics. Mol Phylogenet Evol (2012) 62(2):777–92. doi: 10.1016/j.ympev.2011.11.019 PMC3258026 22138161

[35] Abecasis AB Wensing AMJ Paraskevis D Vercauteren J Theys K Van de Vijver DAMC. HIV-1 subtype distribution and its demographic determinants in newly diagnosed patients in Europe suggest highly compartmentalized epidemics. Retrovirology. (2013) 10(1):7. doi: 10.1186/1742-4690-10-7 23317093 PMC3564855

[36] Souto B Triunfante V Santos-Pereira A Martins J Araújo PMM Osório NS. Evolutionary dynamics of HIV-1 subtype c in Brazil. Sci Rep (2021) 11(1):23060. doi: 10.1038/s41598-021-02428-3 34845263 PMC8629974

[37] Geretti AM. HIV-1 subtypes: epidemiology and significance for HIV management. Curr Opin Infect Dis (2006) 19(1):1–7. doi: 10.1097/01.qco.0000200293.45532.68 16374210

[38] Siddappa NB Dash PK Mahadevan A Desai A Jayasuryan N Ravi V. Identification of unique B/C recombinant strains of HIV-1 in the southern state of karnataka, India. Aids. (2005) 19(13):1426–9. doi: 10.1097/01.aids.0000180795.49016.89 16103776

[39] Soares MA De Oliveira T Brindeiro RM Diaz RS Sabino EC Brigido L. A specific subtype c of human immunodeficiency virus type 1 circulates in Brazil. AIDS. (2003) 17(1):11–21. doi: 10.1097/00002030-200301030-00004 12478065

[40] Bbosa N Kaleebu P Ssemwanga D. HIV Subtype diversity worldwide. Curr Opin HIV AIDS. (2019) 14(3):153–60. doi: 10.1097/COH.0000000000000534 30882484

[41] Riemenschneider M Cashin KY Budeus B Sierra S Shirvani-Dastgerdi E Bayanolhagh S. Genotypic prediction of Co-receptor tropism of HIV-1 subtypes a and c. Sci Rep (2016) 6(1):24883. doi: 10.1038/srep24883 27126912 PMC4850382

[42] Esbjörnsson J Månsson F Martínez-Arias W Vincic E Biague AJ da Silva ZJ. Frequent CXCR4 tropism of HIV-1 subtype a and CRF02_AG during late-stage disease - indication of an evolving epidemic in West Africa. Retrovirology. (2010) 7:23. doi: 10.1186/1742-4690-7-23 20307309 PMC2855529

[43] Ketseoglou I Lukhwareni A Steegen K Carmona S Stevens WS Papathanasopoulos MA. Viral tropism and antiretroviral drug resistance in HIV-1 subtype c-infected patients failing highly active antiretroviral therapy in Johannesburg, south Africa. AIDS Res Hum Retroviruses (2014) 30(3):289–93. doi: 10.1089/aid.2013.0267 PMC3938925 24224886

[44] Cashin K Paukovics G Jakobsen MR Østergaard L Churchill MJ Gorry PR. Differences in coreceptor specificity contribute to alternative tropism of HIV-1 subtype c for CD4+ T-cell subsets, including stem cell memory T-cells. Retrovirology. (2014) 11(1):97. doi: 10.1186/s12977-014-0097-5 25387392 PMC4236466

[45] Ball SC Abraha A Collins KR Marozsan AJ Baird H Quiñones-Mateu ME. Comparing the ex vivo fitness of CCR5-tropic human immunodeficiency virus type 1 isolates of subtypes b and c. J Virol (2003) 77(2):1021–38. doi: 10.1128/JVI.77.2.1021-1038.2003 PMC140829 12502818

[46] Venner CM Nankya I Kyeyune F Demers K Kwok C Chen P-LL. Infecting HIV-1 subtype predicts disease progression in women of Sub-Saharan Africa. EBioMedicine. (2016) 13:305–14. doi: 10.1016/j.ebiom.2016.10.014 PMC5264310 27751765

[47] de Mendoza C Garrido C. Different disease progression rate according to HIV-1 subtype. Futur HIV Ther (2008) 2(4):319–22. doi: 10.2217/17469600.2.4.319

[48] Kanki PJ Hamel DJ Sankalé JL cheng HC Thior I Barin F. Human immunodeficiency virus type 1 subtypes differ in disease progression. J Infect Dis (1999) 179(1):68–73. doi: 10.1086/314557 9841824

[49] Marozsan AJ Moore DM Lobritz MA Fraundorf E Abraha A Reeves JD. Differences in the fitness of two diverse wild-type human immunodeficiency virus type 1 isolates are related to the efficiency of cell binding and entry. J Virol (2005) 79(11):7121–34. doi: 10.1128/JVI.79.11.7121-7134.2005 PMC1112120 15890952

[50] Kaleebu P French N Mahe C Yirrell D Watera C Lyagoba F. Effect of human immunodeficiency virus (HIV) type 1 envelope subtypes a and d on disease progression in a large cohort of HIV-1-positive persons in Uganda. J Infect Dis (2002) 185(9):1244–50. doi: 10.1086/340130 12001041

[51] Lynch RM Shen T Gnanakaran S Derdeyn CA. Appreciating HIV type 1 diversity: subtype differences in env. AIDS Res Hum Retroviruses (2009) 25(3):237–48. doi: 10.1089/aid.2008.0219 PMC2853864 19327047

[52] Gnanakaran S Lang D Daniels M Bhattacharya T Derdeyn CA Korber B. Clade-specific differences between human immunodeficiency virus type 1 clades b and c: diversity and correlations in C3-V4 regions of gp120. J Virol (2007) 81(9):4886–91. doi: 10.1128/JVI.01954-06 PMC1900169 17166900

[53] Derdeyn CA Decker JM Bibollet-Ruche F Mokili JL Muldoon M Denham SA. Envelope-constrained neutralization-sensitive HIV-1 after heterosexual transmission. Sci (80-). (2004) 303(5666):2019–22. doi: 10.1126/science.1093137 15044802

[54] Frost SDW Liu Y Pond SLK Chappey C Wrin T Petropoulos CJ. Characterization of human immunodeficiency virus type 1 (HIV-1) envelope variation and neutralizing antibody responses during transmission of HIV-1 subtype b. J Virol (2005) 79(10):6523–7. doi: 10.1128/JVI.79.10.6523-6527.2005 PMC1091710 15858036

[55] Choisy M Woelk CH Guégan J-F Robertson DL. Comparative study of adaptive molecular evolution in different human immunodeficiency virus groups and subtypes. J Virol (2004) 78(4):1962–70. doi: 10.1128/JVI.78.4.1962-1970.2004 PMC369455 14747561

[56] Patel MB Hoffman NG Swanstrom R. Subtype-specific conformational differences within the V3 region of subtype b and subtype c human immunodeficiency virus type 1 env proteins. J Virol (2008) 82(2):903 LP – 916. doi: 10.1128/JVI.01444-07 PMC2224581 18003735

[57] Travers SAA Connell MJ McCormack GP McInerney JO. Evidence for heterogeneous selective pressures in the evolution of the env gene in different human immunodeficiency virus type 1 subtypes. J Virol (2005) 79(3):1836 LP – 1841. doi: 10.1128/JVI.79.3.1836-1841.2005 PMC544114 15650207

[58] Iordanskiy S Waltke M Feng Y Wood C. Subtype-associated differences in HIV-1 reverse transcription affect the viral replication. Retrovirology. (2010) 7(1):85. doi: 10.1186/1742-4690-7-85 20939905 PMC2964588

[59] Han YS Mesplède T Wainberg MA. Differences among HIV-1 subtypes in drug resistance against integrase inhibitors. Infect Genet Evol (2016) 46:286–91. doi: 10.1016/j.meegid.2016.06.047 27353185

[60] Koning FA Castro H Dunn D Tilston P Cane PA Mbisa JL. Subtype-specific differences in the development of accessory mutations associated with high-level resistance to hiv-1 nucleoside reverse transcriptase inhibitors. J Antimicrob Chemother (2013) 68(6):1220–36. doi: 10.1093/jac/dkt012 23386260

[61] Spira S. Impact of clade diversity on HIV-1 virulence, antiretroviral drug sensitivity and drug resistance. J Antimicrob Chemother (2003) 51(2):229–40. doi: 10.1093/jac/dkg079 12562686

[62] Zazzi M Hu H Prosperi M. The global burden of HIV-1 drug resistance in the past 20 years. PeerJ. (2018) 6:e4848–8. doi: 10.7717/peerj.4848 PMC5971836 29844989

[63] Woodham AW Skeate JG Sanna AM Taylor JR Da Silva DM Cannon PM. Human immunodeficiency virus immune cell receptors, coreceptors, and cofactors: implications for prevention and treatment. AIDS Patient Care STDS. (2016) 30(7):291–306. doi: 10.1089/apc.2016.0100 27410493 PMC4948215

[64] Siliciano JD Siliciano RF. Latency and viral persistence in HIV-1 infection. J Clin Invest. (2000) 106(7):823–5. doi: 10.1172/JCI11246 PMC517817 11018068

[65] Ta TM Malik S Anderson EM Jones AD Perchik J Freylikh M. Insights into persistent HIV-1 infection and functional cure: novel capabilities and strategies. Front Microbiol (2022) 13. doi: 10.3389/fmicb.2022.862270 PMC9093714 35572626

[66] Siliciano RF Greene WC. HIV Latency. Cold Spring Harb Perspect Med (2011) 1(1):a007096. doi: 10.1101/cshperspect.a007096 22229121 PMC3234450

[67] Dufour C Gantner P Fromentin R Chomont N. The multifaceted nature of HIV latency. J Clin Invest. (2020) 130(7):3381–90. doi: 10.1172/JCI136227 PMC7324199 32609095

[68] Brooks K Jones BR Dilernia DA Wilkins DJ Claiborne DT McInally S. HIV-1 variants are archived throughout infection and persist in the reservoir. PloS Pathog (2020) 16(6):e1008378. doi: 10.1371/journal.ppat.1008378 32492044 PMC7295247

[69] Jones BR Kinloch NN Horacsek J Ganase B Harris M Harrigan PR. Phylogenetic approach to recover integration dates of latent HIV sequences within-host. Proc Natl Acad Sci U S A. (2018) 115(38):E8958–67. doi: 10.1073/pnas.1802028115 PMC6156657 30185556

[70] Buzon MJ Martin-Gayo E Pereyra F Ouyang Z Sun H Li JZ. Long-term antiretroviral treatment initiated at primary HIV-1 infection affects the size, composition, and decay kinetics of the reservoir of HIV-1-infected CD4 T cells. J Virol (2014) 88(17):10056–65. doi: 10.1128/JVI.01046-14 PMC4136362 24965451

[71] Ruff CT Ray SC Kwon P Zinn R Pendleton A Hutton N. Persistence of wild-type virus and lack of temporal structure in the latent reservoir for human immunodeficiency virus type 1 in pediatric patients with extensive antiretroviral exposure. J Virol (2002) 76(18):9481–92. doi: 10.1128/JVI.76.18.9481-9492.2002 PMC136462 12186930

[72] Verhofstede C Noë A Demecheleer E De Cabooter N Van Wanzeele F van der Gucht B. Drug-resistant variants that evolve during nonsuppressive therapy persist in HIV-1-infected peripheral blood mononuclear cells after long-term highly active antiretroviral therapy. J Acquir Immune Defic Syndr (2004) 35(5):473–83. doi: 10.1097/00126334-200404150-00005 15021312

[73] Kieffer TL Finucane MM Nettles RE Quinn TC Broman KW Ray SC. Genotypic analysis of HIV-1 drug resistance at the limit of detection: virus production without evolution in treated adults with undetectable HIV loads. J Infect Dis (2004) 189(8):1452–65. doi: 10.1086/382488 15073683

[74] Bailey JR Sedaghat AR Kieffer T Brennan T Lee PK Wind-Rotolo M. Residual human immunodeficiency virus type 1 viremia in some patients on antiretroviral therapy is dominated by a small number of invariant clones rarely found in circulating CD4+ T cells. J Virol (2006) 80(13):6441–57. doi: 10.1128/JVI.00591-06 PMC1488985 16775332

[75] Tsukamoto T. Hematopoietic Stem/Progenitor cells and the pathogenesis of HIV/AIDS. Front Cell Infect Microbiol (2020) 10. doi: 10.3389/fcimb.2020.00060 PMC7047323 32154191

[76] Fan L Li C Zhao H. Prevalence and risk factors of cytopenia in HIV-infected patients before and after the initiation of HAART. BioMed Res Int (2020) 2020:1–10. doi: 10.1155/2020/3132589 PMC7008269 32090076

[77] Gebreweld A Fiseha T Girma N Haileslasie H Gebretsadik D. Prevalence of cytopenia and its associated factors among HIV infected adults on highly active antiretroviral therapy at mehal meda hospital, north shewa zone, Ethiopia. PloS One (2020) 15(9):e0239215. doi: 10.1371/journal.pone.0239215 32931523 PMC7491728

[78] Kyeyune R Saathoff E Ezeamama AE Löscher T Fawzi W Guwatudde D. Prevalence and correlates of cytopenias in HIV-infected adults initiating highly active antiretroviral therapy in Uganda. BMC Infect Dis (2014) 14(1):496. doi: 10.1186/1471-2334-14-496 25209550 PMC4165997

[79] Fiseha T Ebrahim H. Prevalence and predictors of cytopenias in HIV-infected adults at initiation of antiretroviral therapy in mehal meda hospital, central Ethiopia. J Blood Med (2022) 13:201–11. doi: 10.2147/JBM.S355966 PMC9056021 35502291

[80] Kaner J Thibaud S Sridharan A Assal A Polineni R Zingman B. HIV Is associated with a high rate of unexplained multilineage cytopenias and portends a poor prognosis in myelodysplastic syndrome (MDS) and acute myeloid leukemia (AML). Blood. (2016) 128(22):4345–5. doi: 10.1182/blood.V128.22.4345.4345

[81] Sharma S Sachdeva RK Sachdeva MUS Sreedharanunni S Naseem S Sharma P. Bone marrow examination of HIV-infected children in HAART era reveals a spectrum of abnormalities: a study from single tertiary care center of north India. J Hematop. (2021) 14(4):283–90. doi: 10.1007/s12308-021-00471-7

[82] Gunda DW Godfrey KG Kilonzo SB Mpondo BC. Cytopenias among ART-naive patients with advanced HIV disease on enrolment to care and treatment services at a tertiary hospital in Tanzania: a crosssectional study. Malawi Med J (2017) 29(1):43. doi: 10.4314/mmj.v29i1.9 28567196 PMC5442491

[83] Banda NK Simon GR Sipple JD Terrell KL Archer P Shpall EJ. Depletion of CD34+CD4+ cells in bone marrow from HIV-1-infected individuals. Biol Blood Marrow Transplant. (1999) 5(3):162–72. doi: 10.1053/bbmt.1999.v5.pm10392962 10392962

[84] Shah I Murthy A. Bone marrow abnormalities in HIV infected children, report of three cases and review of the literature. J Res Med Sci (2014) 19(2):181–3. PMC3999606 24778674

[85] Koka P Reddy S. Cytopenias in HIV infection: mechanisms and alleviation of hematopoietic inhibition. Curr HIV Res (2004) 2(3):275–82. doi: 10.2174/1570162043351282 15279591

[86] Alexaki A Wigdahl B. HIV-1 infection of bone marrow hematopoietic progenitor cells and their role in trafficking and viral dissemination. PloS Pathog (2008) 4(12):e1000215. doi: 10.1371/journal.ppat.1000215 19112504 PMC2603331

[87] Wong ME Jaworowski A Hearps AC. The HIV reservoir in monocytes and macrophages. Front Immunol (2019) 10(JUN). doi: 10.3389/fimmu.2019.01435 PMC6607932 31297114

[88] Maryanovich M Takeishi S Frenette PS. Neural regulation of bone and bone marrow. Cold Spring Harb Perspect Med (2018) 8(9):a031344. doi: 10.1101/cshperspect.a031344 29500307 PMC6119651

[89] Yamazaki S Ema H Karlsson G Yamaguchi T Miyoshi H Shioda S. Nonmyelinating schwann cells maintain hematopoietic stem cell hibernation in the bone marrow niche. Cell. (2011) 147(5):1146–58. doi: 10.1016/j.cell.2011.09.053 22118468

[90] Datta G Miller NM Afghah Z Geiger JD Chen X. HIV-1 gp120 promotes lysosomal exocytosis in human schwann cells. Front Cell Neurosci (2019) 13. doi: 10.3389/fncel.2019.00329 PMC6650616 31379513

[91] Xu X Zheng L Yuan Q Zhen G Crane JL Zhou X. Transforming growth factor-β in stem cells and tissue homeostasis. Bone Res (2018) 6(1):2. doi: 10.1038/s41413-017-0005-4 29423331 PMC5802812

[92] Mahadevan A Gayathri N Taly AB Santosh V Yasha TC Shankar SK. Vasculitic neuropathy in HIV infection: a clinicopathological study. Neurol India. (2001) 49(3):277–83. 11593246

[93] Keswani SC Polley M Pardo CA Griffin JW McArthur JC Hoke A. Schwann cell chemokine receptors mediate HIV-1 gp120 toxicity to sensory neurons. Ann Neurol (2003) 54(3):287–96. doi: 10.1002/ana.10645 12953261

[94] Ntogwa M Imai S Hiraiwa R Koyanagi M Matsumoto M Ogihara T. Schwann cell-derived CXCL1 contributes to human immunodeficiency virus type 1 gp120-induced neuropathic pain by modulating macrophage infiltration in mice. Brain Behav Immun (2020) 88:325–39. doi: 10.1016/j.bbi.2020.03.027 32229220

[95] Renders S Svendsen AF Panten J Rama N Maryanovich M Sommerkamp P. Niche derived netrin-1 regulates hematopoietic stem cell dormancy *via* its receptor neogenin-1. Nat Commun (2021) 12(1):608. doi: 10.1038/s41467-020-20801-0 33504783 PMC7840807

[96] Xu C Gao X Wei Q Nakahara F Zimmerman SE Mar J. Stem cell factor is selectively secreted by arterial endothelial cells in bone marrow. Nat Commun (2018) 9(1):2449. doi: 10.1038/s41467-018-04726-3 29934585 PMC6015052

[97] Ding L Saunders TL Enikolopov G Morrison SJ. Endothelial and perivascular cells maintain haematopoietic stem cells. Nature. (2012) 481(7382):457–62. doi: 10.1038/nature10783 PMC3270376 22281595

[98] Kenswil KJG Jaramillo AC Ping Z Chen S Hoogenboezem RM Mylona MA. Characterization of endothelial cells associated with hematopoietic niche formation in humans identifies IL-33 as an anabolic factor. Cell Rep (2018) 22(3):666–78. doi: 10.1016/j.celrep.2017.12.070 29346765

[99] Almeida-Porada G Ascensāo JL. Isolation, characterization, and biologic features of bone marrow endothelial cells. J Lab Clin Med (1996) 128(4):399–407. doi: 10.1016/S0022-2143(96)80012-6 8833889

[100] Li WM Huang WQ Huang YH De Jiang Z Wang QR. Positive and negative haematopoietic cytokines produced by bone marrow endothelial cells. Cytokine. (2000) 12(7):1017–23. doi: 10.1006/cyto.1999.0678 10880247

[101] Moses AV Williams S Heneveld ML Strussenberg J Rarick M Loveless M. Human immunodeficiency virus infection of bone marrow endothelium reduces induction of stromal hematopoietic growth factors. Blood. (1996) 87(3):919–25. doi: 10.1182/blood.V87.3.919.bloodjournal873919 8562963

[102] Herrmann CH Rice AP. Specific interaction of the human immunodeficiency virus tat proteins with a cellular protein kinase. Virology. (1993) 197(2):601–8. doi: 10.1006/viro.1993.1634 8249283

[103] Zidovetzki R Wang JL Chen P Jeyaseelan R Hofman F. Human immunodeficiency virus tat protein induces interleukin 6 mRNA expression in human brain endothelial cells *via* protein kinase c- and cAMP- dependent protein kinaes pathways. AIDS Res Hum Retroviruses (1998) 14(10):825–33. doi: 10.1089/aid.1998.14.825 9671211

[104] Kamtchum-Tatuene J Mwandumba H Al-Bayati Z Flatley J Griffiths M Solomon T. HIV Is associated with endothelial activation despite ART, in a sub-Saharan African setting. Neurol - Neuroimmunol Neuroinflammation. (2019) 6(2):e531. doi: 10.1212/NXI.0000000000000531 PMC6340379 30697583

[105] Dhawan S Puri RK Kumar A Duplan H Masson JM Aggarwal BB. Human immunodeficiency virus-1-tat protein induces the cell surface expression of endothelial leukocyte adhesion molecule-1, vascular cell adhesion molecule-1, and intercellular adhesion molecule-1 in human endothelial cells. Blood. (1997) 90(4):1535–44. doi: 10.1182/blood.V90.4.1535 9269771

[106] Hofman FM Wright AD Dohadwala MM Wong-Staal F Walker SM. Exogenous tat protein activates human endothelial cells. Blood. (1993) 82(9):2774–80. doi: 10.1182/blood.V82.9.2774.2774 7693046

[107] Acheampong EA Parveen Z Muthoga LW Kalayeh M Mukhtar M Pomerantz RJ. Human immunodeficiency virus type 1 nef potently induces apoptosis in primary human brain microvascular endothelial cells *via* the activation of caspases. J Virol (2005) 79(7):4257–69. doi: 10.1128/JVI.79.7.4257-4269.2005 PMC1061575 15767427

[108] Qi M Aiken C. Nef enhances HIV-1 infectivity *via* association with the virus assembly complex. Virology. (2008) 373(2):287–97. doi: 10.1016/j.virol.2007.12.001 PMC2440657 18191978

[109] Wang T Green LA Gupta SK Kim C Wang L Almodovar S. Transfer of intracellular HIV nef to endothelium causes endothelial dysfunction. PloS One (2014) 9(3):e91063. doi: 10.1371/journal.pone.0091063 24608713 PMC3946685

[110] Vilhardt F Plastre O Sawada M Suzuki K Wiznerowicz M Kiyokawa E. The HIV-1 nef protein and phagocyte NADPH oxidase activation. J Biol Chem (2002) 277(44):42136–43. doi: 10.1074/jbc.M200862200 12207012

[111] Duffy P Wang X Lin PH Yao Q Chen C. HIV Nef protein causes endothelial dysfunction in porcine pulmonary arteries and human pulmonary artery endothelial cells. J Surg Res (2009) 156(2):257–64. doi: 10.1016/j.jss.2009.02.005 PMC2760402 19540523

[112] Caccuri F Giagulli C Bugatti A Benetti A Alessandri G Ribatti D. HIV-1 matrix protein p17 promotes angiogenesis *via* chemokine receptors CXCR1 and CXCR2. Proc Natl Acad Sci (2012) 109(36):14580–5. doi: 10.1073/pnas.1206605109 PMC3437870 22904195

[113] Marini E Tiberio L Caracciolo S Tosti G Guzman CA Schiaffonati L. HIV-1 matrix protein p17 binds to monocytes and selectively stimulates MCP-1 secretion: role of transcriptional factor AP-1. Cell Microbiol (2008) 10(3):655–66. doi: 10.1111/j.1462-5822.2007.01073.x PMC7162350 18042260

[114] Mazzuca P Caruso A Caccuri F. Endothelial cell dysfunction in HIV-1 Infection. In: Endothelial dysfunction - old concepts and new challenges. (Rijeka: InTech) (2018). doi: 10.5772/intechopen.73023

[115] Aoki K Kurashige M Ichii M Higaki K Sugiyama T Kaito T. Identification of CXCL12-abundant reticular cells in human adult bone marrow. Br J Haematol (2021) 193(3):659–68. doi: 10.1111/bjh.17396 PMC8252541 33837967

[116] Sugiyama T Kohara H Noda M Nagasawa T. Maintenance of the hematopoietic stem cell pool by CXCL12-CXCR4 chemokine signaling in bone marrow stromal cell niches. Immunity. (2006) 25(6):977–88. doi: 10.1016/j.immuni.2006.10.016 17174120

[117] Semerad CL Christopher MJ Liu F Short B Simmons PJ Winkler I. G-CSF potently inhibits osteoblast activity and CXCL12 mRNA expression in the bone marrow. Blood. (2005) 106(9):3020–7. doi: 10.1182/blood-2004-01-0272 PMC1895331 16037394

[118] Möhle R Bautz F Rafii S Moore MA Brugger W Kanz L. The chemokine receptor CXCR-4 is expressed on CD34+ hematopoietic progenitors and leukemic cells and mediates transendothelial migration induced by stromal cell-derived factor-1. Blood. (1998) 91(12):4523–30. doi: 10.1182/blood.V91.12.4523.412k04_4523_4530 9616148

[119] Schajnovitz A Itkin T D’Uva G Kalinkovich A Golan K Ludin A. CXCL12 secretion by bone marrow stromal cells is dependent on cell contact and mediated by connexin-43 and connexin-45 gap junctions. Nat Immunol (2011) 12(5):391–8. doi: 10.1038/ni.2017 21441933

[120] Abe-Suzuki S Kurata M Abe S Onishi I Kirimura S Nashimoto M. CXCL12+ stromal cells as bone marrow niche for CD34+ hematopoietic cells and their association with disease progression in myelodysplastic syndromes. Lab Investig (2014) 94(11):1212–23. doi: 10.1038/labinvest.2014.110 25199050

[121] Ding J Zhao J Zhou J Li X Wu Y Ge M. Association of gene polymorphism of SDF1(CXCR12) with susceptibility to HIV-1 infection and AIDS disease progression: a meta-analysis. PloS One (2018) 13(2):e0191930. doi: 10.1371/journal.pone.0191930 29420545 PMC5805253

[122] Zhang J Supakorndej T Krambs JR Rao M Abou-Ezzi G Ye RY. Bone marrow dendritic cells regulate hematopoietic stem/progenitor cell trafficking. J Clin Invest. (2019) 129(7):2920–31. doi: 10.1172/JCI124829 PMC6597218 31039135

[123] Clayton KL Garcia JV Clements JE Walker BD. Hiv infection of macrophages: implications for pathogenesis and cure. Pathog Immun (2017) 2(2):179–92. doi: 10.20411/pai.v2i2.204 PMC5526341 28752134

[124] Hendricks CM Cordeiro T Gomes AP Stevenson M. The interplay of HIV-1 and macrophages in viral persistence. Front Microbiol (2021) 12. doi: 10.3389/fmicb.2021.646447 PMC8058371 33897659

[125] Veenhuis RT Abreu CM Costa PAG Ferreira EA Ratliff J Pohlenz L. Monocyte-derived macrophages contain persistent latent HIV reservoirs. Nat Microbiol (2023) 8(5):833–44. doi: 10.1038/s41564-023-01349-3 PMC10159852 36973419

[126] Sakaguchi M Sato T Groopman JE. Human immunodeficiency virus infection of megakaryocytic cells. Blood. (1991) 77(3):481–5. doi: 10.1182/blood.V77.3.481.481 1991165

[127] Sato T Sekine H Kakuda H Miura N Sunohara M Fuse A. HIV Infection of megakaryocytic cell lines. Leuk Lymphoma. (2000) 36(3–4):397–404. doi: 10.3109/10428190009148861 10674912

[128] Voulgaropoulou F Pontow SE Ratner L. Productive infection of CD34+-Cell-Derived megakaryocytes by X4 and R5 HIV-1 isolates. Virology. (2000) 269(1):78–85. doi: 10.1006/viro.2000.0193 10725200

[129] Zucker-Franklin D Cao YZ. Megakaryocytes of human immunodeficiency virus-infected individuals express viral RNA. Proc Natl Acad Sci U S A. (1989) 86(14):5595–9. doi: 10.1073/pnas.86.14.5595 PMC297669 2748605

[130] Chen K Jiao Y Liu L Huang M He C He W. Communications between bone marrow macrophages and bone cells in bone remodeling. Front Cell Dev Biol (2020) 8. doi: 10.3389/fcell.2020.598263 PMC7783313 33415105

[131] Ehninger A Trumpp A. The bone marrow stem cell niche grows up: mesenchymal stem cells and macrophages move in. J Exp Med (2011) 208(3):421–8. doi: 10.1084/jem.20110132 PMC3058583 21402747

[132] Sims NA Quinn JMW. Osteoimmunology: oncostatin m as a pleiotropic regulator of bone formation and resorption in health and disease. Bonekey Rep (2014) 3. doi: 10.1038/bonekey.2014.22 PMC4037876 24876928

[133] Crayne CB Albeituni S Nichols KE Cron RQ. The immunology of macrophage activation syndrome. Front Immunol (2019) 10. doi: 10.3389/fimmu.2019.00119 PMC6367262 30774631

[134] Iyer SS Cheng G. Role of interleukin 10 transcriptional regulation in inflammation and autoimmune disease. Crit Rev Immunol (2012) 32(1):23–63. doi: 10.1615/CritRevImmunol.v32.i1.30 22428854 PMC3410706

[135] Maggini J Mirkin G Bognanni I Holmberg J Piazzón IM Nepomnaschy I. Mouse bone marrow-derived mesenchymal stromal cells turn activated macrophages into a regulatory-like profile. Neyrolles O editor. PloS One (2010) 5(2):e9252. doi: 10.1371/journal.pone.0009252 PMC2821929 20169081

[136] Winkler IG Sims NA Pettit AR Barbier V Nowlan B Helwani F. Bone marrow macrophages maintain hematopoietic stem cell (HSC) niches and their depletion mobilizes HSCs. Blood. (2010) 116(23):4815–28. doi: 10.1182/blood-2009-11-253534 20713966

[137] Chow A Lucas D Hidalgo A Méndez-Ferrer S Hashimoto D Scheiermann C. Bone marrow CD169+ macrophages promote the retention of hematopoietic stem and progenitor cells in the mesenchymal stem cell niche. J Exp Med (2011) 208(2):261–71. doi: 10.1084/jem.20101688 PMC3039855 21282381

[138] Canque B Marandin A Rosenzwajg M Louache F Vainchenker W Gluckman JC. Susceptibility of human bone marrow stromal cells to human immunodeficiency virus (HIV). Virology. (1995) 208(2):779–83. doi: 10.1006/viro.1995.1211 7747451

[139] Campbell JH Hearps AC Martin GE Williams KC Crowe SM. The importance of monocytes and macrophages in HIV pathogenesis, treatment, and cure. AIDS. (2014) 28(15):2175–87. doi: 10.1097/QAD.0000000000000408 PMC6331181 25144219

[140] Araínga M Edagwa B Mosley RL Poluektova LY Gorantla S Gendelman HE. A mature macrophage is a principal HIV-1 cellular reservoir in humanized mice after treatment with long acting antiretroviral therapy. Retrovirology. (2017) 14(1):17. doi: 10.1186/s12977-017-0344-7 28279181 PMC5345240

[141] Ladinsky MS Khamaikawin W Jung Y Lin S Lam J An DS. Mechanisms of virus dissemination in bone marrow of HIV-1–infected humanized BLT mice. Elife (2019) 8. doi: 10.7554/eLife.46916 PMC6839903 31657719

[142] Honeycutt JB Wahl A Baker C Spagnuolo RA Foster J Zakharova O. Macrophages sustain HIV replication *in vivo* independently of T cells. J Clin Invest. (2016) 126(4):1353–66. doi: 10.1172/JCI84456 PMC4811134 26950420

[143] Guo L Xu X-Q Zhou L Zhou R-H Wang X Li J-L. Human intestinal epithelial cells release antiviral factors that inhibit HIV infection of macrophages. Front Immunol (2018) 9. doi: 10.3389/fimmu.2018.00247 PMC5825896 29515574

[144] Xu X-Q Guo L Wang X Liu Y Liu H Zhou R-H. Human cervical epithelial cells release antiviral factors and inhibit HIV replication in macrophages. J Innate Immun (2019) 11(1):29–40. doi: 10.1159/000490586 30032138 PMC6338329

[145] Zhang B Liu J Zhou L Wang X Khan S Hu W. Cytosolic DNA sensor activation inhibits HIV infection of macrophages. J Med Virol (2023) 95(1). doi: 10.1002/jmv.28253 PMC9839519 36286245

[146] Denu RA Nemcek S Bloom DD Goodrich AD Kim J Mosher DF. Fibroblasts and mesenchymal Stromal/Stem cells are phenotypically indistinguishable. Acta Haematol (2016) 136(2):85–97. doi: 10.1159/000445096 27188909 PMC4988914

[147] Haniffa MA Collin MP Buckley CD Dazzi F. Mesenchymal stem cells: the fibroblasts’ new clothes? Haematologica (2009) 94(2):258–63. doi: 10.3324/haematol.13699 PMC2635401 19109217

[148] Soundararajan M Kannan S. Fibroblasts and mesenchymal stem cells: two sides of the same coin? J Cell Physiol (2018) 233(12):9099–109. doi: 10.1002/jcp.26860 29943820

[149] Dorronsoro A Lang V Ferrin I Fernández-Rueda J Zabaleta L Pérez-Ruiz E. Intracellular role of IL-6 in mesenchymal stromal cell immunosuppression and proliferation. Sci Rep (2020) 10(1):21853. doi: 10.1038/s41598-020-78864-4 33318571 PMC7736882

[150] Pricola KL Kuhn NZ Haleem-Smith H Song Y Tuan RS. Interleukin-6 maintains bone marrow-derived mesenchymal stem cell stemness by an ERK1/2-dependent mechanism. J Cell Biochem (2009) 108(3):577–88. doi: 10.1002/jcb.22289 PMC2774119 19650110

[151] Starc N Ingo D Conforti A Rossella V Tomao L Pitisci A. Biological and functional characterization of bone marrow-derived mesenchymal stromal cells from patients affected by primary immunodeficiency. Sci Rep (2017) 7(1):8153. doi: 10.1038/s41598-017-08550-5 28811575 PMC5557950

[152] Frisch BJ Porter RL Gigliotti BJ Olm-Shipman AJ Weber JM O’Keefe RJ. *In vivo* prostaglandin E2 treatment alters the bone marrow microenvironment and preferentially expands short-term hematopoietic stem cells. Blood. (2009) 114(19):4054–63. doi: 10.1182/blood-2009-03-205823 PMC2774547 19726721

[153] Porter RL Georger MA Bromberg O McGrath KE Frisch BJ Becker MW. Prostaglandin E2 increases hematopoietic stem cell survival and accelerates hematopoietic recovery after radiation injury. Stem Cells (2013) 31(2):372–83. doi: 10.1002/stem.1286 PMC3580384 23169593

[154] Porter RL Calvi LM. Prostaglandin E2 is rapidly produced in response to bone marrow injury and improves survival of primitive hematopoietic cells. Blood. (2010) 116(21):407–7. doi: 10.1182/blood.V116.21.407.407

[155] Dolgalev I Tikhonova AN. Connecting the dots: resolving the bone marrow niche heterogeneity. Front Cell Dev Biol (2021) 9. doi: 10.3389/fcell.2021.622519 PMC7994602 33777933

[156] Mabuchi Y Okawara C Méndez-Ferrer S Akazawa C. Cellular heterogeneity of mesenchymal Stem/Stromal cells in the bone marrow. Front Cell Dev Biol (2021) 9. doi: 10.3389/fcell.2021.689366 PMC8291416 34295894

[157] Zhou BO Yu H Yue R Zhao Z Rios JJ Naveiras O. Bone marrow adipocytes promote the regeneration of stem cells and haematopoiesis by secreting SCF. Nat Cell Biol (2017) 19(8):891–903. doi: 10.1038/ncb3570 28714970 PMC5536858

[158] Czechowicz A Kraft D Weissman IL Bhattacharya D. Efficient transplantation *via* antibody-based clearance of hematopoietic stem cell niches. Sci (80-). (2007) 318(5854):1296–9. doi: 10.1126/science.1149726 PMC2527021 18033883

[159] Wang Z Li X Yang J Gong Y Zhang H Qiu X. Single-cell RNA sequencing deconvolutes the *in vivo* heterogeneity of human bone marrow-derived mesenchymal stem cells. Int J Biol Sci (2021) 17(15):4192–206. doi: 10.7150/ijbs.61950 PMC8579438 34803492

[160] Kallmeyer K Ryder MA Pepper MS. Mesenchymal stromal cells: a possible reservoir for HIV-1? Stem Cell Rev Rep (2022) 18(4):1253–80. doi: 10.1007/s12015-021-10298-5 PMC9033703 34973144

[161] Gibellini D Alviano F Miserocchi A Tazzari PL Ricci F Clò A. HIV-1 and recombinant gp120 affect the survival and differentiation of human vessel wall-derived mesenchymal stem cells. Retrovirology. (2011) 8(1):40. doi: 10.1186/1742-4690-8-40 21612582 PMC3123274

[162] Scadden DT Zeira M Woon A Wang Z Schieve L Ikeuchi K. Human immunodeficiency virus infection of human bone marrow stromal fibroblasts. Blood. (1990) 76(2):317–22. doi: 10.1182/blood.V76.2.317.317 1695109

[163] Wang L Mondal D La Russa VF Agrawal KC. Suppression of clonogenic potential of human bone marrow mesenchymal stem cells by HIV type 1: putative role of HIV type 1 tat protein and inflammatory cytokines. AIDS Res Hum Retroviruses (2002) 18(13):917–31. doi: 10.1089/088922202760265597 12230935

[164] Beaupere C Garcia M Larghero J Fève B Capeau J Lagathu C. The HIV proteins tat and nef promote human bone marrow mesenchymal stem cell senescence and alter osteoblastic differentiation. Aging Cell (2015) 14(4):534–46. doi: 10.1111/acel.12308 PMC4531068 25847297

[165] Cotter EJ Ip HSM Powderly WG Doran PP. Mechanism of HIV protein induced modulation of mesenchymal stem cell osteogenic differentiation. BMC Musculoskelet Disord (2008) 9(1):33. doi: 10.1186/1471-2474-9-33 18366626 PMC2330047

[166] Cotter EJ Malizia AP Chew N Powderly WG Doran PP. HIV Proteins regulate bone marker secretion and transcription factor activity in cultured human osteoblasts with consequent potential implications for osteoblast function and development. AIDS Res Hum Retroviruses (2007) 23(12):1521–9. doi: 10.1089/aid.2007.0112 18160010

[167] Weivoda MM Chew CK Monroe DG Farr JN Atkinson EJ Geske JR. Identification of osteoclast-osteoblast coupling factors in humans reveals links between bone and energy metabolism. Nat Commun (2020) 11(1):87. doi: 10.1038/s41467-019-14003-6 31911667 PMC6946812

[168] Blank U Karlsson S. TGF-β signaling in the control of hematopoietic stem cells. Blood. (2015) 125(23):3542–50. doi: 10.1182/blood-2014-12-618090 25833962

[169] Galán-Díez M Kousteni S. The osteoblastic niche in hematopoiesis and hematological myeloid malignancies. Curr Mol Biol Rep (2017) 3(2):53–62. doi: 10.1007/s40610-017-0055-9 29098141 PMC5662025

[170] Adams GB Chabner KT Alley IR Olson DP Szczepiorkowski ZM Poznansky MC. Stem cell engraftment at the endosteal niche is specified by the calcium-sensing receptor. Nature. (2006) 439(7076):599–603. doi: 10.1038/nature04247 16382241

[171] Arai F Hirao A Ohmura M Sato H Matsuoka S Takubo K. Tie2/angiopoietin-1 signaling regulates hematopoietic stem cell quiescence in the bone marrow niche. Cell. (2004) 118(2):149–61. doi: 10.1016/j.cell.2004.07.004 15260986

[172] Yoshihara H Arai F Hosokawa K Hagiwara T Takubo K Nakamura Y. Thrombopoietin/MPL signaling regulates hematopoietic stem cell quiescence and interaction with the osteoblastic niche. Cell Stem Cell (2007) 1(6):685–97. doi: 10.1016/j.stem.2007.10.020 18371409

[173] Gohda J Ma Y Huang Y Zhang Y Gu L Han Y. HIV-1 replicates in human osteoclasts and enhances their differentiation *in vitro* . Retrovirology (2015) 12(1):12. doi: 10.1186/s12977-015-0139-7 25809599 PMC4340110

[174] Raynaud-Messina B Bracq L Dupont M Souriant S Usmani SM Proag A. Bone degradation machinery of osteoclasts: an HIV-1 target that contributes to bone loss. Proc Natl Acad Sci U S A. (2018) 115(11):E2556–65. doi: 10.1073/pnas.1713370115 PMC5856515 29463701

[175] Delpino MV Quarleri J. Influence of HIV infection and antiretroviral therapy on bone homeostasis. Front Endocrinol (Lausanne). (2020) 11:11. doi: 10.3389/fendo.2020.00502 32982960 PMC7493215

[176] Mansour A Abou-Ezzi G Sitnicka E Jacobsen SEW Wakkach A Blin-Wakkach C. Osteoclasts promote the formation of hematopoietic stem cell niches in the bone marrow. J Exp Med (2012) 209(3):537–49. doi: 10.1084/jem.20110994 PMC3302238 22351931

[177] Xie M Leroy H Mascarau R Woottum M Dupont M Ciccone C. Cell-to-Cell spreading of HIV-1 in myeloid target cells escapes SAMHD1 restriction. MBio. (2019) 10(6):e02457–19. doi: 10.1128/mBio.02457-19 PMC6867896 31744918

[178] Nacher M Serrano S González A Hernández A Mariñoso ML Vilella R. Osteoblasts in HIV-infected patients: HIV-1 infection and cell function. Aids. (2001) 15(17):2239–43. doi: 10.1097/00002030-200111230-00004 11698696

[179] Venhoff N Walker UA. Pathogenesis of bone disorders in HIV infection. Int J Clin Rheumtol. (2009) 4(2):147–59. doi: 10.2217/ijr.09.10

[180] Cummins NW Klicpera A Sainski AM Bren GD Khosla S Westendorf JJ. Human immunodeficiency virus envelope protein Gp120 induces proliferation but not apoptosis in osteoblasts at physiologic concentrations. PloS One (2011) 6(9):e24876–6. doi: 10.1371/journal.pone.0024876 PMC3171487 21931863

[181] Gibellini D De Crignis E Ponti C Cimatti L Borderi M Tschon M. HIV-1 triggers apoptosis in primary osteoblasts and HOBIT cells through TNFα activation. J Med Virol (2008) 80(9):1507–14. doi: 10.1002/jmv.21266 18649336

[182] Tang Y Wu X Lei W Pang L Wan C Shi Z. TGF-beta1-induced migration of bone mesenchymal stem cells couples bone resorption with formation. Nat Med (2009) 15(7):757–65. doi: 10.1038/nm.1979 PMC2727637 19584867

[183] Challen GA Boles NC Chambers SM Goodell MA. Distinct hematopoietic stem cell subtypes are differentially regulated by TGF-β1. Cell Stem Cell (2010) 6(3):265–78. doi: 10.1016/j.stem.2010.02.002 PMC2837284 20207229

[184] Lima A. Osteopenia and osteoporosis in people living with HIV: multiprofessional approach. HIV/AIDS - Res Palliat Care (2011) 117:117–24. doi: 10.2147/HIV.S6617 PMC3257973 22267944

[185] Kruger MJ Nell TA. Bone mineral density in people living with HIV: a narrative review of the literature. AIDS Res Ther (2017) 14(1):35. doi: 10.1186/s12981-017-0162-y 28747190 PMC5530558

[186] McComsey GA Tebas P Shane E Yin MT Overton ET Huang JS. Bone disease in HIV infection: a practical review and recommendations for HIV care providers. Clin Infect Dis (2010) 51(8):937–46. doi: 10.1086/656412 PMC3105903 20839968

[187] Wakabayashi Y Yoshino Y Seo K Koga I Kitazawa T Ota Y. Inhibition of osteoblast differentiation by ritonavir. BioMed Rep (2018) 9(6):491–6. doi: 10.3892/br.2018.1154 PMC6256180 30546876

[188] Bergamaschi A Pancino G. Host hindrance to HIV-1 replication in monocytes and macrophages. Retrovirology. (2010) 7(1):31. doi: 10.1186/1742-4690-7-31 20374633 PMC2868797

[189] Izquierdo-Useros N Lorizate M McLaren PJ Telenti A Kräusslich H-G Martinez-Picado J. HIV-1 capture and transmission by dendritic cells: the role of viral glycolipids and the cellular receptor siglec-1. PloS Pathog (2014) 10(7):e1004146. doi: 10.1371/journal.ppat.1004146 25033082 PMC4102576

[190] Ahmed Z Kawamura T Shimada S Piguet V. The role of human dendritic cells in HIV-1 infection. J Invest Dermatol (2015) 135(5):1225–33. doi: 10.1038/jid.2014.490 25407434

[191] Manches O Frleta D Bhardwaj N. Dendritic cells in progression and pathology of HIV infection. Trends Immunol (2014) 35(3):114–22. doi: 10.1016/j.it.2013.10.003 PMC3943663 24246474

[192] Schmidt B Ashlock BM Foster H Fujimura SH Levy JA. HIV-Infected cells are major inducers of plasmacytoid dendritic cell interferon production, maturation, and migration. Virology. (2005) 343(2):256–66. doi: 10.1016/j.virol.2005.09.059 16278001

[193] Lepelley A Louis S Sourisseau M Law HKW Pothlichet J Schilte C. Innate sensing of HIV-infected cells. Emerman M editor. PloS Pathog (2011) 7(2):e1001284. doi: 10.1371/journal.ppat.1001284 PMC3040675 21379343

[194] Martín-Moreno A Muñoz-Fernández MA. Dendritic cells, the double agent in the war against HIV-1. Front Immunol (2019) 10. doi: 10.3389/fimmu.2019.02485 PMC6820366 31708924

[195] Wu L KewalRamani VN. Dendritic-cell interactions with HIV: infection and viral dissemination. Nat Rev Immunol (2006) 6(11):859–68. doi: 10.1038/nri1960 PMC1796806 17063186

[196] Ziegler SM Altfeld M. Human immunodeficiency virus 1 and type I interferons–where sex makes a difference. Front Immunol (2017) 8:8. doi: 10.3389/fimmu.2017.01224 29033943 PMC5625005

[197] Murira A Lamarre A. Type-I interferon responses: from friend to foe in the battle against chronic viral infection. Front Immunol (2016) 7. doi: 10.3389/fimmu.2016.00609 PMC5165262 28066419

[198] Pietras EM Lakshminarasimhan R Techner J-M Fong S Flach J Binnewies M. Re-entry into quiescence protects hematopoietic stem cells from the killing effect of chronic exposure to type I interferons. J Exp Med (2014) 211(2):245–62. doi: 10.1084/jem.20131043 PMC3920566 24493802

[199] Demerdash Y Kain B Essers MAG King KY. Yin and yang: the dual effects of interferons on hematopoiesis. Exp Hematol (2021) 96:1–12. doi: 10.1016/j.exphem.2021.02.002 33571568 PMC8919039

[200] Huot N Bosinger SE Paiardini M Reeves RK Müller-Trutwin M. Lymph node cellular and viral dynamics in natural hosts and impact for HIV cure strategies. Front Immunol (2018) 9:780. doi: 10.3389/fimmu.2018.00780 29725327 PMC5916971

[201] Chun TW Stuyver L Mizell SB Ehler LA Mican JAM Baseler M. Presence of an inducible HIV-1 latent reservoir during highly active antiretroviral therapy. Proc Natl Acad Sci U S A. (1997) 94(24):13193–7. doi: 10.1073/pnas.94.24.13193 PMC24285 9371822

[202] Chen JJ-Y Huang JC Shirtliff M Briscoe E Ali S Cesani F. CD4 lymphocytes in the blood of HIV(+) individuals migrate rapidly to lymph nodes and bone marrow: support for homing theory of CD4 cell depletion. J Leukoc Biol (2002) 72(2):271–8. doi: 10.1189/jlb.72.2.271 12149417

[203] Reuter MA Pombo C Betts MR. Cytokine production and dysregulation in HIV pathogenesis: lessons for development of therapeutics and vaccines. Cytokine Growth Factor Rev (2012) 23(4–5):181–91. doi: 10.1016/j.cytogfr.2012.05.005 PMC3582023 22743036

[204] Gao A Gong Y Zhu C Yang W Li Q Zhao M. Bone marrow endothelial cell-derived interleukin-4 contributes to thrombocytopenia in acute myeloid leukemia. Haematologica. (2019) 104(10):1950–61. doi: 10.3324/haematol.2018.214593 PMC6886411 30792200

[205] Okhrimenko A Grün JR Westendorf K Fang Z Reinke S von Roth P. Human memory T cells from the bone marrow are resting and maintain long-lasting systemic memory. Proc Natl Acad Sci (2014) 111(25):9229–34. doi: 10.1073/pnas.1318731111 PMC4078840 24927527

[206] Chahroudi A Silvestri G Lichterfeld M. T Memory stem cells and HIV: a long-term relationship. Curr HIV/AIDS Rep (2015) 12(1):33–40. doi: 10.1007/s11904-014-0246-4 25578055 PMC4370789

[207] Di Rosa F Gebhardt T. Bone marrow T cells and the integrated functions of recirculating and tissue-resident memory T cells. Front Immunol (2016) 7. doi: 10.3389/fimmu.2016.00051 PMC4754413 26909081

[208] Di Rosa F Pabst R. The bone marrow: a nest for migratory memory T cells. Trends Immunol (2005) 26(7):360–6. doi: 10.1016/j.it.2005.04.011 15978522

[209] Di Rosa F. Maintenance of memory T cells in the bone marrow: survival or homeostatic proliferation? Nat Rev Immunol (2016) 16(4):271–1. doi: 10.1038/nri.2016.31 26996200

[210] Zhao E Xu H Wang L Kryczek I Wu K Hu Y. Bone marrow and the control of immunity. Cell Mol Immunol (2012) 9(1):11–9. doi: 10.1038/cmi.2011.47 PMC3251706 22020068

[211] Sebastian NT Collins KL. Targeting HIV latency: resting memory T cells, hematopoietic progenitor cells and future directions. Expert Rev Anti Infect Ther (2014) 12(10):1187–201. doi: 10.1586/14787210.2014.956094 PMC4291319 25189526

[212] Hoang TN Harper JL Pino M Wang H Micci L King CT. Bone marrow-derived CD4 + T cells are depleted in simian immunodeficiency virus-infected macaques and contribute to the size of the replication-competent reservoir. J Virol (2019) 93(1). doi: 10.1128/JVI.01344-18 PMC6288341 30305357

[213] Carter CC Onafuwa-Nuga A McNamara LA Riddell J Bixby D Savona MR. HIV-1 infects multipotent progenitor cells causing cell death and establishing latent cellular reservoirs. Nat Med (2010) 16(4):446–51. doi: 10.1038/nm.2109 PMC2892382 20208541

[214] Nixon CC Vatakis DN Reichelderfer SN Dixit D Kim SG Uittenbogaart CH. HIV-1 infection of hematopoietic progenitor cells *in vivo* in humanized mice. Blood. (2013) 122(13):2195–204. doi: 10.1182/blood-2013-04-496950 PMC3785119 23886835

[215] Zou W Xing J Wang F Chen X Liu Q Wang J. HIV-1LAI nef blocks the development of hematopoietic stem/progenitor cells into T lymphoid cells. AIDS. (2021) 35(6):851–60. doi: 10.1097/QAD.0000000000002837 PMC8048728 33587447

[216] Stanley SK Kessler SW Justement JS Schnittman SM Greenhouse JJ Brown CC. CD34+ bone marrow cells are infected with HIV in a subset of seropositive individuals. J Immunol (1992) 149(2):689–97. doi: 10.4049/jimmunol.149.2.689 1378076

[217] Neal TF Holland HK Baum CM Villinger F Ansari AA Saral R. CD34+ progenitor cells from asymptomatic patients are not a major reservoir for human immunodeficiency virus-1. Blood. (1995) 86(5):1749–56. doi: 10.1182/blood.V86.5.1749.bloodjournal8651749 7544640

[218] Redd AD Avalos A Essex M. Infection of hematopoietic progenitor cells by HIV-1 subtype c, and its association with anemia in southern Africa. Blood. (2007) 110(9):3143–9. doi: 10.1182/blood-2007-04-086314 PMC2200905 17693583

[219] Durand CM Ghiaur G Siliciano JD Rabi SA Eisele EE Salgado M. HIV-1 DNA is detected in bone marrow populations containing CD4 + T cells but is not found in purified CD34 + hematopoietic progenitor cells in most patients on antiretroviral therapy. J Infect Dis (2012) 205(6):1014–8. doi: 10.1093/infdis/jir884 PMC3282572 22275402

[220] Josefsson L Eriksson S Sinclair E Ho T Killian M Epling L. Hematopoietic precursor cells isolated from patients on long-term suppressive HIV therapy did not contain HIV-1 DNA. J Infect Dis (2012) 206(1):28–34. doi: 10.1093/infdis/jis301 22536001 PMC3415927

[221] Griffin DO Goff SP. HIV-1 is restricted prior to integration of viral DNA in primary cord-derived human CD34 + cells. J Virol (2015) 89(15):8096–100. doi: 10.1128/JVI.01044-15 PMC4505644 25995256

[222] Renelt S Schult-Dietrich P Baldauf H-M Stein S Kann G Bickel M. HIV-1 infection of long-lived hematopoietic precursors in vitro and *In vivo* . Cells (2022) 11(19):2968. doi: 10.3390/cells11192968 36230931 PMC9562211

[223] Molina J Scadden D Sakaguchi M Fuller B Woon A Groopman J. Lack of evidence for infection of or effect on growth of hematopoietic progenitor cells after *in vivo* or *in vitro* exposure to human immunodeficiency virus. Blood. (1990) 76(12):2476–82. doi: 10.1182/blood.V76.12.2476.2476 2265244

[224] Davis BR Schwartz DH Marx JC Johnson CE Berry JM Lyding J. Absent or rare human immunodeficiency virus infection of bone marrow stem/progenitor cells in vivo. J Virol (1991) 65(4):1985–90. doi: 10.1128/jvi.65.4.1985-1990.1991 PMC240035 2002553

[225] Louache F Henri A Bettaieb A Oksenhendler E Raguin G Tulliez M. Role of human immunodeficiency virus replication in defective *in vitro* growth of hematopoietic progenitors. Blood. (1992) 80(12):2991–9. doi: 10.1182/blood.V80.12.2991.2991 1281682

[226] Chelucci C Hassan HJ Locardi C Bulgarini D Pelosi E Mariani G. *In vitro* human immunodeficiency virus-1 infection of purified hematopoietic progenitors in single-cell culture. Blood. (1995) 85(5):1181–7. doi: 10.1182/blood.V85.5.1181.bloodjournal8551181 7532032

[227] Marandin A Katz A Oksenhendler E Tulliez M Picard F Vainchenker W. Loss of primitive hematopoietic progenitors in patients with human immunodeficiency virus infection. Blood. (1996) 88(12):4568–78. doi: 10.1182/blood.V88.12.4568.bloodjournal88124568 8977248

[228] Weichold FF Zella D Barabitskaja O Maciejewski JP Dunn DE Sloand EM. Neither human immunodeficiency virus-1 (HIV-1) nor HIV-2 infects most-primitive human hematopoietic stem cells as assessed in long-term bone marrow cultures. Blood. (1998) 91(3):907–15. doi: 10.1182/blood.V91.3.907 9446651

[229] Shen H Cheng T Preffer FI Dombkowski D Tomasson MH Golan DE. Intrinsic human immunodeficiency virus type 1 resistance of hematopoietic stem cells despite coreceptor expression. J Virol (1999) 73(1):728–37. doi: 10.1128/JVI.73.1.728-737.1999 PMC103880 9847379

[230] Carter CC McNamara LA Onafuwa-Nuga A Shackleton M Riddell IVJ Bixby D. HIV-1 utilizes the CXCR4 chemokine receptor to infect multipotent hematopoietic stem and progenitor cells. Cell Host Microbe (2011) 9(3):223–34. doi: 10.1016/j.chom.2011.02.005 PMC3102232 21402361

[231] Zhang J Scadden DT Crumpacker CS. Primitive hematopoietic cells resist HIV-1 infection *via* p21 Waf1/Cip1/Sdi1. J Clin Invest. (2007) 117(2):473–81. doi: 10.1172/JCI28971 PMC1783820 17273559

[232] Bordoni V Bibas M Abbate I Viola D Rozera G Agrati C. Bone marrow CD34+ progenitor cells may harbour HIV-DNA even in successfully treated patients. Clin Microbiol Infect (2015) 21(3):290.e5–8. doi: 10.1016/j.cmi.2014.11.003 25658531

[233] Araínga M Su H Poluektova LY Gorantla S Gendelman HE. HIV-1 cellular and tissue replication patterns in infected humanized mice. Sci Rep (2016) 6:23513. doi: 10.1038/srep23513 26996968 PMC4800734

[234] Zaikos TD Terry VH Sebastian Kettinger NT Lubow J Painter MM Virgilio MC. Hematopoietic stem and progenitor cells are a distinct HIV reservoir that contributes to persistent viremia in suppressed patients. Cell Rep (2018) 25(13):3759–3773.e9. doi: 10.1016/j.celrep.2018.11.104 30590047

[235] McNamara LA Ganesh JA Collins KL. Latent HIV-1 infection occurs in multiple subsets of hematopoietic progenitor cells and is reversed by NF-kappaB activation. J Virol (2012) 86(17):9337–50. doi: 10.1128/JVI.00895-12 PMC3416176 22718820

[236] McNamara LA Onafuwa-Nuga A Sebastian NT Riddell J4 Bixby D Collins KL. CD133+ hematopoietic progenitor cells harbor HIV genomes in a subset of optimally treated people with long-term viral suppression. J Infect Dis (2013) 207(12):1807–16. doi: 10.1093/infdis/jit118 PMC3654754 23554378

[237] Sebastian NT Zaikos TD Terry V Taschuk F McNamara LA Onafuwa-Nuga A. CD4 is expressed on a heterogeneous subset of hematopoietic progenitors, which persistently harbor CXCR4 and CCR5-tropic HIV proviral genomes *in vivo* . PloS Pathog (2017) 13(7):e1006509. doi: 10.1371/journal.ppat.1006509 28732051 PMC5540617

[238] Liu Y Liu H Kim BO Gattone VH Li J Nath A. CD4-independent infection of astrocytes by human immunodeficiency virus type 1: requirement for the human mannose receptor. J Virol (2004) 78(8):4120–33. doi: 10.1128/JVI.78.8.4120-4133.2004 PMC374297 15047828

[239] Saha K Zhang J Gupta A Dave R Yimen M Zerhouni B. Isolation of primary HIV-1 that target CD8+ T lymphocytes using CD8 as a receptor. Nat Med (2001) 7(1):65–72. doi: 10.1038/83365 11135618

[240] Xiao P Usami O Suzuki Y Ling H Shimizu N Hoshino H. Characterization of a CD4-independent clinical HIV-1 that can efficiently infect human hepatocytes through chemokine (C-X-C motif) receptor 4. Aids. (2008) 22(14):1749–57. doi: 10.1097/QAD.0b013e328308937c 18753859

[241] Yoshii H Kamiyama H Goto K Oishi K Katunuma N Tanaka Y. CD4-independent human immunodeficiency virus infection involves participation of endocytosis and cathepsin b. PloS One (2011) 6(4):e19352. doi: 10.1371/journal.pone.0019352 21541353 PMC3081840

[242] Skinner AM O’Neill SL Grompe M Kurre P. CXCR4 induction in hematopoietic progenitor cells from fanca–/–, -c–/–, and -d2–/– mice. Exp Hematol (2008) 36(3):273–82. doi: 10.1016/j.exphem.2007.11.006 PMC2335150 18279715

[243] Stevenson M Stanwick TL Dempsey MP Lamonica CA. HIV-1 replication is controlled at the level of T cell activation and proviral integration. EMBO J (1990) 9(5):1551–60. doi: 10.1002/j.1460-2075.1990.tb08274.x PMC551849 2184033

[244] Sumide K Matsuoka Y Kawamura H Nakatsuka R Fujioka T Asano H. A revised road map for the commitment of human cord blood CD34-negative hematopoietic stem cells. Nat Commun (2018) 9(1):2202. doi: 10.1038/s41467-018-04441-z 29875383 PMC5989201

[245] Griffin DO Goff SP. Restriction of HIV-1-based lentiviral vectors in adult primary marrow-derived and peripheral mobilized human CD34+ hematopoietic stem and progenitor cells occurs prior to viral DNA integration. Retrovirology. (2016) 13(1):14. doi: 10.1186/s12977-016-0246-0 26945863 PMC4779582

[246] Cilliers T Nhlapo J Coetzer M Orlovic D Ketas T Olson WC. The CCR5 and CXCR4 coreceptors are both used by human immunodeficiency virus type 1 primary isolates from subtype c. J Virol (2003) 77(7):4449–56. doi: 10.1128/JVI.77.7.4449-4456.2003 PMC150635 12634405

[247] Baeten JM Chohan B Lavreys L Chohan V McClelland RS Certain L. HIV-1 subtype d infection is associated with faster disease progression than subtype a in spite of similar plasma HIV-1 loads. J Infect Dis (2007) 195(8):1177–80. doi: 10.1086/512682 17357054

[248] Desfosses Y Solis M Sun Q Grandvaux N Van Lint C Burny A. Regulation of human immunodeficiency virus type 1 gene expression by clade-specific tat proteins. J Virol (2005) 79(14):9180–91. doi: 10.1128/JVI.79.14.9180-9191.2005 PMC1168763 15994812

[249] Rogers L Obasa AE Jacobs GB Sarafianos SG Sönnerborg A Neogi U. Structural implications of genotypic variations in HIV-1 integrase from diverse subtypes. Front Microbiol (2018) 9:1754. doi: 10.3389/fmicb.2018.01754 30116231 PMC6083056

[250] Roof P Ricci M Genin P Montano MA Essex M Wainberg MA. Differential regulation of HIV-1 clade-specific b, c, and e long terminal repeats by NF-kappaB and the tat transactivator. Virology. (2002) 296(1):77–83. doi: 10.1006/viro.2001.1397 12036319

[251] Izopet J Tamalet C Pasquier C Sandres K Marchou B Massip P. Quantification of HIV-1 proviral DNA by a standardized colorimetric PCR-based assay. J Med Virol (1998) 54(1):54–9. doi: 10.1002/(SICI)1096-9071(199801)54:1<54::AID-JMV8>3.0.CO;2-O 9443109

[252] Brinchmann JE Albert J Vartdal F. Few infected CD4+ T cells but a high proportion of replication-competent provirus copies in asymptomatic human immunodeficiency virus type 1 infection. J Virol (1991) 65(4):2019–23. doi: 10.1128/jvi.65.4.2019-2023.1991 PMC240046 1672165

[253] Josefsson L King MS Makitalo B Brännström J Shao W Maldarelli F. Majority of CD4 + T cells from peripheral blood of HIV-1-infected individuals contain only one HIV DNA molecule. Proc Natl Acad Sci U S A. (2011) 108(27):11199–204. doi: 10.1073/pnas.1107729108 PMC3131354 21690402

[254] Psallidopoulos MC Schnittman SM Thompson 3LM Baseler M Fauci AS HC L. Integrated proviral human immunodeficiency virus type 1 is present in CD4+ peripheral blood lymphocytes in healthy seropositive individuals. J Virol (1989) 63(11):4626–31. doi: 10.1128/jvi.63.11.4626-4631.1989 PMC251096 2795714

[255] Zauli G Vitale M Gibellini D Capitani S. Inhibition of purified CD34+ hematopoietic progenitor cells by human immunodeficiency virus 1 or gp120 mediated by endogenous transforming growth factor beta 1. J Exp Med (1996) 183(1):99–108. doi: 10.1084/jem.183.1.99 8551249 PMC2192418

[256] Rameshwar P Denny TN Gascón P. Enhanced HIV-1 activity in bone marrow can lead to myelopoietic suppression partially contributed by gag p24. J Immunol (1996) 157(9):4244–50. doi: 10.4049/jimmunol.157.9.4244 8892663

[257] Kulkosky J Laptev A Shetty S Srinivasan A BouHamdan M Prockop DJ. Human immunodeficiency virus type 1 vpr alters bone marrow cell function. Blood. (1999) 93(6):1906–15. doi: 10.1182/blood.V93.6.1906.406k11_1906_1915 10068663

[258] Gibellini D Vitone F Buzzi M Schiavone P De Crignis E Cicola R. HIV-1 negatively affects the survival/maturation of cord blood CD34+ hematopoietic progenitor cells differentiated towards megakaryocytic lineage by HIV-1 gp120/CD4 membrane interaction. J Cell Physiol (2007) 210(2):315–24. doi: 10.1002/jcp.20815 17111363

[259] Prost S Le Dantec M Augé S Le Grand R Derdouch S Auregan G. Human and simian immunodeficiency viruses deregulate early hematopoiesis through a Nef/PPARgamma/STAT5 signaling pathway in macaques. J Clin Invest. (2008) 118(5):1765–75. doi: 10.1172/JCI33037 PMC2323187 18431514

